# PHGDH inhibition and FOXO3 modulation drives PUMA-dependent apoptosis in osteosarcoma

**DOI:** 10.1038/s41419-025-07378-6

**Published:** 2025-02-12

**Authors:** Toshinao Oyama, Caitlyn B. Brashears, Richa Rathore, Heather Benect-Hamilton, Katharine E. Caldwell, Naomi Dirckx, William G. Hawkins, Brian A. Van Tine

**Affiliations:** 1 Department of Medicine, Division of Medical Oncology, Washington University in St. Louis, St. Louis, MO USA; 2 Department of Surgery, Division of Hepatobiliary Surgery, Washington University in St. Louis, St. Louis, MO USA; 3 Department of Orthopedics, Washington University in St. Louis, St. Louis, MO USA; 4 Siteman Cancer Center, St. Louis, MO USA; 5 Department of Pediatric Hematology/Oncology, St Louis Children’s Hospital, St Louis, MO USA

**Keywords:** Bone cancer, Sarcoma, Apoptosis

## Abstract

Osteosarcoma is a bone cancer that has been found to be metabolically dependent on the conversion of glucose to serine through the rate-limiting enzyme 3-phosphoglycerate dehydrogenase (PHGDH). The upregulation of PHGDH has been correlated with poor patient survival, and the inhibition of the serine synthesis pathway using targeted small-molecule inhibition of PHGDH induces a rapid metabolic adaptation that prevents cell death due to pro-survival signaling through the mammalian target of rapamycin complex 1 (mTORC1) pathway. Here, PHGDH inhibition in combination with mTORC1 signaling modulation for the treatment of osteosarcoma was evaluated. When combined with PHGDH inhibition, several non-rapalog inhibitors of mTORC1 activated Forkhead box O (FOXO) transcription factor 3 (FOXO3), a transcription factor associated with various cellular processes driving apoptosis. The activation of FOXO3 led to transcriptional activation of the pro-apoptotic gene p53 upregulated modulator of apoptosis (PUMA), inducing apoptosis when combined with PHGDH inhibition. These data suggest a path for the clinical development of PHGDH inhibitors in conjunction with mTORC1 pathway modulators in osteosarcoma.

## Introduction

Osteosarcoma (OS) is the most common primary malignant bone tumor in both children and adults, with a propensity to metastasize to the lungs [1–3]. The current standard chemotherapy for osteosarcoma is a combination of doxorubicin, cisplatin, and high-dose methotrexate (HD-MTX) [4]. HD-MTX inhibits dihydrofolate reductase (DHFR), an enzyme in the folate cycle that is essential for the synthesis of DNA, RNA, certain amino acids, and homocysteine metabolism [5]. Although HD-MTX treatment impairs DNA replication and induces cell death, the required dosage is toxic and can cause mucositis, myelosuppression, hepatotoxicity, and nephrotoxicity, particularly in adults. Despite this toxicity, this regimen has been used for over 40 years [6, 7].

Given the relevance of DHFR inhibition in OS therapy, previous research has focused on the folate cycle and upstream pathways [7]. This research has shown that OS relies heavily on the serine biosynthetic pathway, which converts glycolytic intermediates into the amino acid serine. This pathway is essential for protein and nucleotide synthesis, and serves as a precursor for glycine and cysteine. The enzyme 3-phosphoglycerate dehydrogenase (PHGDH) is the rate-limiting step in this pathway, catalyzing the oxidation of 3-phosphoglycerate (3-PG) to 3-phosphohydroxypyruvate (3-PHP). High PHGDH expression is observed in various cancers and is associated with poor prognosis, including lower relapse-free and overall survival rates [8, 9]. Consequently, PHGDH inhibition has been evaluated as a therapeutic strategy, and several PHGDH inhibitors have been developed [10–16].

PHGDH inhibition in OS attenuates cellular proliferation without inducing cell death due to the accumulation of S-adenosylmethionine (SAM) and methionine, subsequently activating the mammalian target of rapamycin (mTOR) pathway as a pro-survival mechanism [8]. This inhibition also upregulates genes associated with the mTOR, AMP-activated protein kinase (AMPK), phosphoinositide 3-kinase (PI3K), and mitogen-activated protein kinase (MAPK) pathways [17]. Combined inhibition of PHGDH with the inhibitor NCT-503 and mTORC1 with the non-rapalog mTOR inhibitor perhexiline, but not rapamycin, induces cell death in OS cells [17]. However, the underlying mechanism behind this effect remains unclear.

Perhexiline, originally developed as an anti-anginal drug in the 1970s, is an mTORC1 inhibitor [18] and also has been identified as an AMPK activator [19]. However, the mechanism of action of perhexiline remains unclear. AMPK is a highly conserved sensor molecule of cellular nutrients and energy status central to regulating energy homeostasis [20]. AMPK is a heterotrimeric complex composed of a catalytic α subunit and regulatory β and γ subunits, and the phosphorylation of tyrosine 172 in the α subunit’s activation loop is crucial for AMPK’s full activation [21–23].

The PI3K-AKT pathway is frequently dysregulated and hyperactivated in cancers, contributing to tumorigenesis and progression. Akt, a key serine/threonine kinase in this pathway, is activated by 3-phosphoinositide-dependent kinase 1 (PDK1) and mTORC2 through phosphorylation at Threonine 308 and Serine 473, respectively [24]. In OS, common alterations in this pathway include mutations in PIK3CA, which encodes the catalytic subunit of PI3K, or the loss of PTEN [25]. These genetic alterations result in persistent Akt hyperactivation, which indirectly activates mTORC1 by phosphorylating the Tuberous Sclerosis Complex (TSC) at Serine 939 and Threonine 1462 [26]. Conversely, AMPK represses mTORC1 by phosphorylating TSC2 at Serine 1387 [27].

Forkhead box O (FOXO) transcription factors belong to the forkhead family [28]. In mammals, the FOXO family includes FOXO1, FOXO3, FOXO4, and FOXO6, which share similar structure, function, and regulation [28]. FOXO1 and 3 play crucial roles in regulating various cellular processes including apoptosis, cell cycle progression, oxidative stress resistance, and metabolism [29]. AKT phosphorylation leads to cytoplasmic retention and inactivation of FOXO1/3, whereas AMPK phosphorylation results in nuclear translocation and gene promotion, including the regulation of apoptosis [30] [31].

To address why combined inhibition of PHGDH with NCT-503 and the inhibition of mTORC1 with perhexiline, but not rapamycin, leads to synergistic cell death in OS, another non-rapalog mTORC1 inhibitor, ALPI3MT55, was employed. Here, we report the mechanism of PHGDH inhibitor resistance as both the activation of AKT and the inhibition of AMPK pathways. Furthermore, this resistance mechanism was bypassed, and cell death was achieved through the combined inhibition of PHGDH, and the inhibition of AKT by ALPI3MT55, or activation of AMPK by perhexiline. Synergy is seen in the combination of all three pathways, where signaling converges at FOXO3 inducing PUMA dependent apoptosis. Notably, the combination of AKT inhibition and AMPK activation induced cell death without PHGDH inhibition. Adding NCT-503 to this combination further promotes apoptosis in OS, allowing for the first step in clinical translation of a three-drug therapy.

## Results

### Inhibition of PHGDH suppressed cell proliferation without inducing cell death in OS

**Fig. 1:**
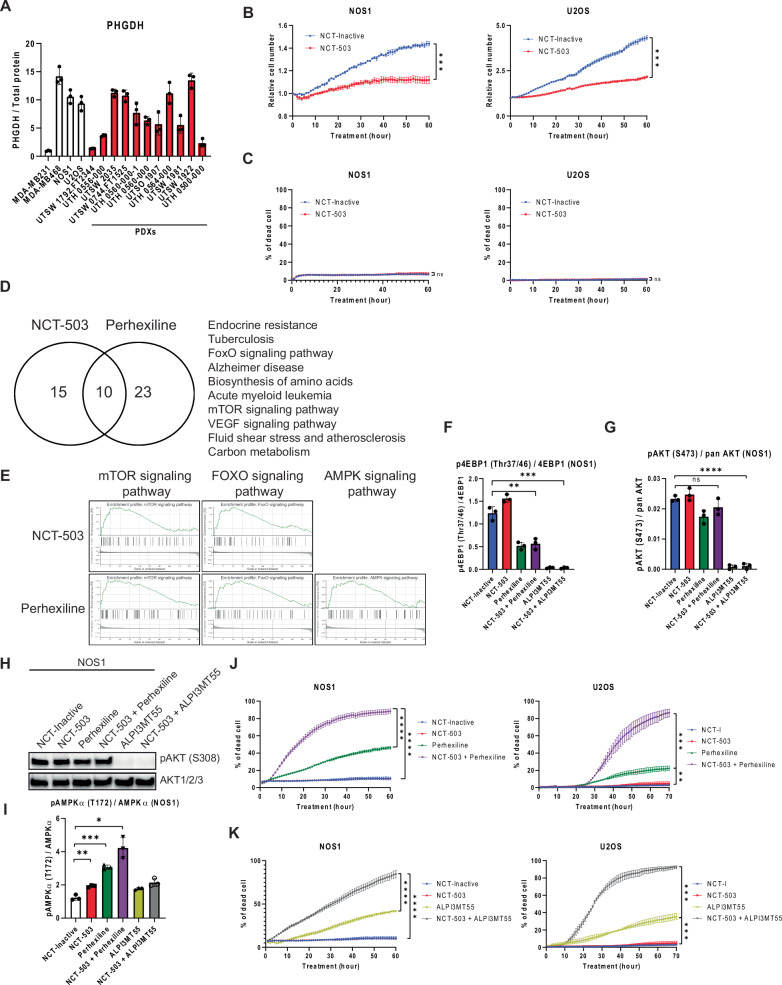
Combined inhibition of PHGDH and non rapalog mTORC1 inhibitors induces cell death of OS cells. **A** PHGDH expression in OS cell lines and OS PDXs. MDA-MB231 and MB468 are shown as the negative and positive control, respectively. Cell proliferation (**B**) and cell death (**C**) of OS cells under PHGDH inhibition. **D** Venn diagram between the results of GSEA using KEGG database on OS cells treated with NCT-503 or perhexiline. Ten overlapping pathways were shown among the enriched KEGG pathways in the NCT-503 and perhexiline treatments. **E** GSEA of mTOR, FOXO on OS cells treated with NCT-503 or perhexiline, and AMPK signaling pathways under perhexiline treatment. **F** Measurement of phosphorylation of 4EBP1 in NOS1 cells under the combined treatment of NCT-503 and either perhexiline or ALPI3MT55 using WES. Phosphorylation of AKT at Ser473 (**G**) and Ser308 (**H**) in NOS1 cells under the combined treatment of NCT-503, perhexiline, and ALPI3MT55. **I** Phosphorylation of AMPKα in NOS1 cells treated with NCT-503, perhexiline, and ALPI3MT55. The cell death of OS cells under combined inhibition of PHGDH and mTORC1 with non-rapalog mTORC1 inhibitor perhexiline (**J**) or ALPI3MT55 (**K**). All experiments are *n* = 3 at least. Bars represent means of values; error bars represent SEM. *P < 0.05, **P < 0.01, ***p < 0.005, ****p < 0.001.

The non-essential amino acid serine plays a critical role in cancer cell survival and proliferation. Using the PHGDH negative cell line MDA-MB231 and the PHGDH high expressing cell line MDA-MB486 for comparison, OS cell lines and a panel of patient-derived xenografts (PDXs) exhibited high expression levels of PHGDH (Fig. 1A). Consistent with prior reports in OS and breast cancer [17], inhibition of PHGDH with NCT-503 suppressed the proliferation of OS cells without induction of cell death (Fig. 1B, C, Supplementary Fig. 1A, B). To identify the mechanisms of cell death induction by the combination treatment of perhexiline and NCT-503, OS cells were treated with either perhexiline or NCT-503 (Supplementary Tables 1), and NanoString metabolism gene panel profiling was performed. Gene set enrichment analysis (GSEA) was conducted using Kyoto Encyclopedia of Genes and Genomes (KEGG) database and 10 overlapping pathways were identified (Fig. 1D). Notably, both the NCT-503 and the perhexiline treatment affected mTOR and FOXO signaling pathways in addition to serine amino acid synthesis (Fig. 1D, E). In addition, perhexiline treatment significantly upregulated AMPK pathway related genes expression (Fig. 1D, E, Supplementary Fig. 1C).

To gain further insight into the mechanisms underlying the cell death mechanism induced by the combination of PHGDH inhibition and mTORC1 by non-rapalog mTORC1 inhibitors, the potent PI3K/mTORC1 inhibitor ALPI3MT55 was employed. To investigate the effect of non-rapalog inhibitors perhexiline and AMPI3MT55 on mTORC1, AMPK and PI3K-AKT pathways, NOS1 cells were treated with either a combination of NCT-503 and perhexiline, NCT-503 and ALPI3MT55, or the single agents; and the phosphorylation of 4EBP1 [32], AKT [24], and AMPK [20] was quantified. Both perhexiline and ALPI3MT55 significantly repressed the phosphorylation of 4EBP1 when combined with NCT-503 but not with the structurally related inactive form of NCT-503 (NCT-Inactive) [33] (Fig. 1F). ALPI3MT55, but not perhexiline, also inhibited the phosphorylation of AKT at Ser473 caused by the mTORC2 complex (Fig. 1G). Additionally, ALPI3MT55 inhibited the phosphorylation of AKT at Ser308, which is mediated by PDK1 (Fig. 1H). Perhexiline treatment led to an increase in the phosphorylation of AMPKα at Thr172 (Fig. 1I), which is an activating phosphorylation. NCT-503 single treatment also increased the phosphorylation of AMPKα, and the combined treatment of NCT-503 with perhexiline resulted in a greater increase in AMPKα phosphorylation as compared to perhexiline treatment alone (Fig. 1I). The individual treatments of either perhexiline or ALPI3MT55 on OS cells induced cell death, and each of these non-raplog inhibitors significantly enhanced cell death when combined with NCT-503 in the OS cell lines, NOS1 and U2OS (Fig. 1J, K). In contrast, despite the significant suppression of mTORC1 activity by rapamycin, NCT-503 treatment did not result in further induction of cell death when combined with rapamycin in OS cells (Supplementary Fig. 1D, E).

### NCT-503 in combination with non-rapalog mTORC1 inhibitors induced apoptotic cell death in OS cells

**Fig. 2:**
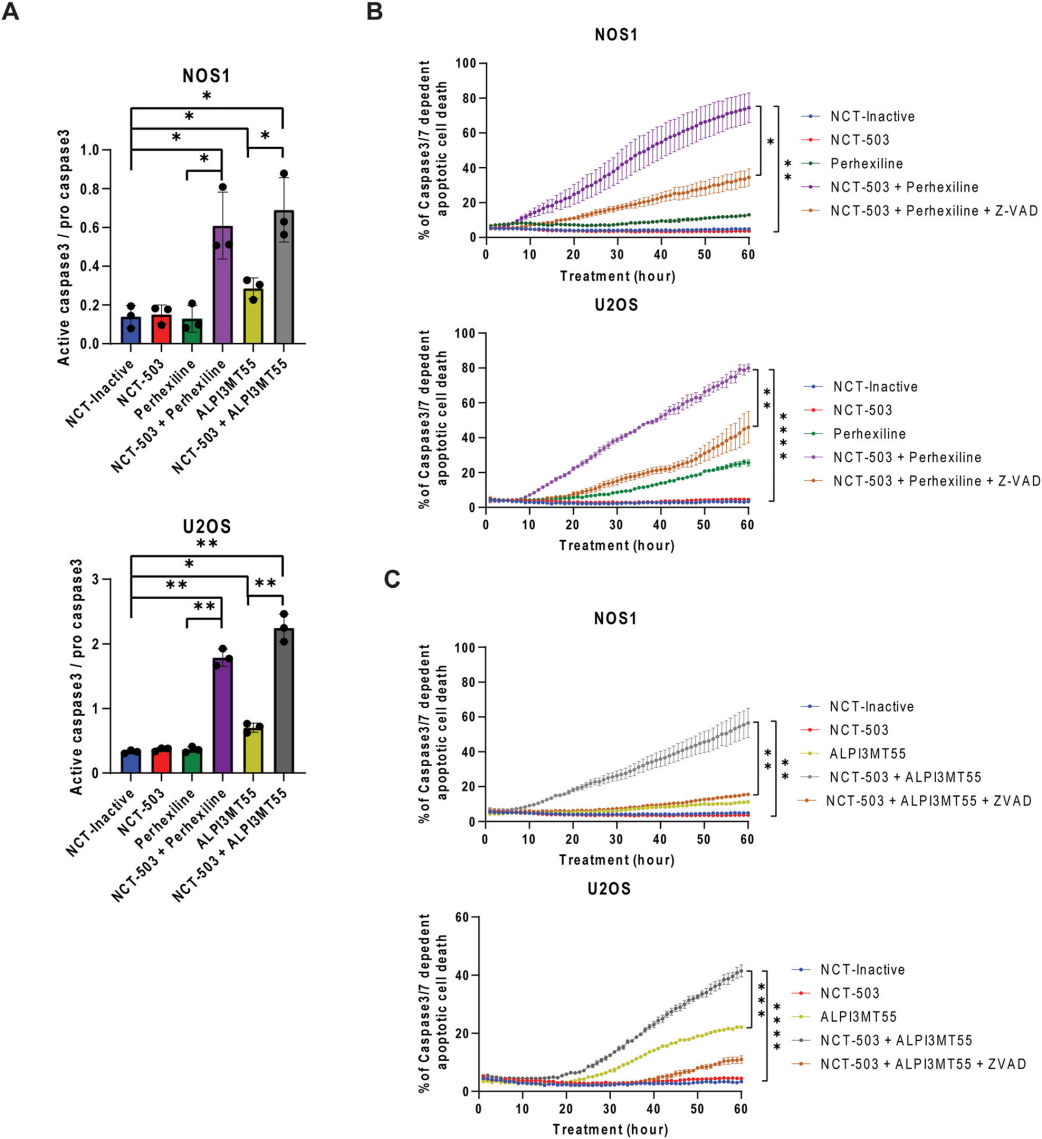
Both combined treatment of NCT-503 and perhexiline/ALPI3MT55 lead to apoptotic cell death. **A** Expression of cleaved caspase 3 under the combined treatments in OS cells. **B** Perhexiline treatment induced capase3/7-dependent apoptotic cell death with PHGDH inhibition, and pan caspase inhibitor Z-VAD-FMK repressed the cell death induction caused by the combined treatment. **C** ALPI3MT55 also induced apoptosis with PHGDH inhibition in OS cells. All experiments are *n* = 3 at least. Bars represent means of values; error bars represent SEM. *P < 0.05, **P < 0.01, ***p < 0.005, ****p < 0.001.

To determine the type of cell death induced by the combined treatment of NCT-503 and non-rapalog mTORC1 inhibitors, cleaved Caspase 3 was quantified. OS cells treated with either NCT-503 or perhexiline did not exhibit increased cleavage of Caspase 3. However, the combined treatment of NCT-503 and perhexiline significantly increased Caspase 3 cleavage (Fig. 2A). OS cell lines treated with ALPI3MT55 alone showed a slight increase in Caspase 3 cleavage, and this increase became significant when ALPI3MT55 was combined with NCT-503 (Fig. 2A). Additionally, OS cell lines treated with NCT-503 combined with either perhexiline or ALPI3MT55 exhibited significant cleaved Caspase 3/7-dependent cell death (Fig. 2B). The pan-Caspase inhibitor, Z-VAD-FMK, suppressed this induction of cell death, suggesting that the type of cell death induced was apoptosis. (Fig. 2B, C).

### NCT-503 in combination with perhexiline or ALPI3MT55 leads to transcriptional activation of *PUMA* and repression of MCL1 protein expression

**Fig. 3:**
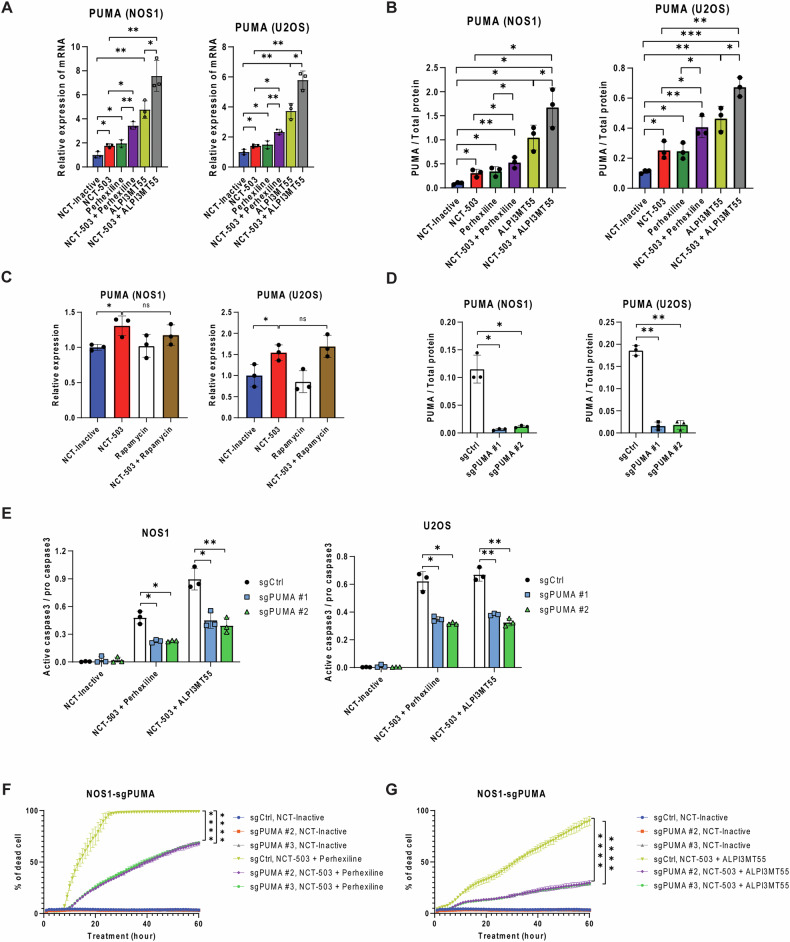
Combined inhibition of PHGDH and mTORC1 with non-rapalog mTORC1 inhibitors elevate the transcription of the pro-apoptotic gene *PUMA.* **A** The mRNA expression of *PUMA* in OS cells under the combined treatments. **B** Protein expression of PUMA under the combined treatments. **C** Rapamycin treatment did not promote *PUMA* transcription of OS cells with NCT-503. **D** Protein expression of PUMA in *PUMA* KO OS cells. **E** Expression of cleaved caspase 3 protein in *PUMA* KO OS cells under the combined treatment. Percentage of cell death of *PUMA* KO OS cells under the combined treatment of NCT-503 and perhexiline (**F**) and the combined treatment of NCT-503 and ALPI3MT55 (**G**). All experiments are *n* = 3 at least. Bars represent means of values; error bars represent SEM. *P < 0.05, **P < 0.01, ***p < 0.005, ****p < 0.001.

To identify the key regulatory molecules involved in the apoptosis induced by the combined treatments in OS cells, changes in the expression levels of apoptosis regulatory genes were investigated. The treatment of OS cells with NCT-503, perhexiline, or ALPI3MT55 upregulated the transcription of pro-apoptotic genes PUMA, NOXA, and BIM (Fig. 3A, Supplementary Fig. 2A). Additionally, NCT-503, when combined with either perhexiline or ALPI3MT55, led to further upregulation of these genes (Fig. 3A, Supplementary Fig. 2A). Notably, the expression of PUMA increased significantly under all treatment conditions (Fig. 3A). The combination treatment of NCT-503 with either perhexiline or ALPI3MT55 increased PUMA protein expression (Fig. 3B). In contrast, the combined treatment of NCT-503 with rapamycin did not lead to the transcriptional activation of PUMA in OS cells (Fig. 3C). To determine the dependence of PUMA induction on the apoptosis induced by the combined treatment, PUMA knockout (KO) was performed in OS cells (Fig. 3D) and the apoptosis induced by the combined treatment was evaluated. The deletion of PUMA in OS cells reduced the cleavage of Caspase 3 and cell death caused by the combined treatments (Fig. 3E–G).

MCL1 is an anti-apoptotic member of the BCL-2 family, and its expression is regulated by mTOR at the translational level, contributing to cell survival and proliferation [34]. To evaluate the impact of mTOR inhibition by the non-rapalog mTORC1 inhibitors on apoptosis induction, MCL1 expression was quantified. MCL1 protein expression was reduced under non-rapalog inhibitor treatments (Supplementary Fig. 2B) without changes in MCL1 mRNA expression (Supplementary Fig. 2C). Additionally, rapamycin treatment also repressed MCL1 protein expression in OS cells (Supplementary Fig. 2D).

### NCT-503 treatment of OS cells leads to increased AMP/ATP ratios and AMPK activation

**Fig. 4:**
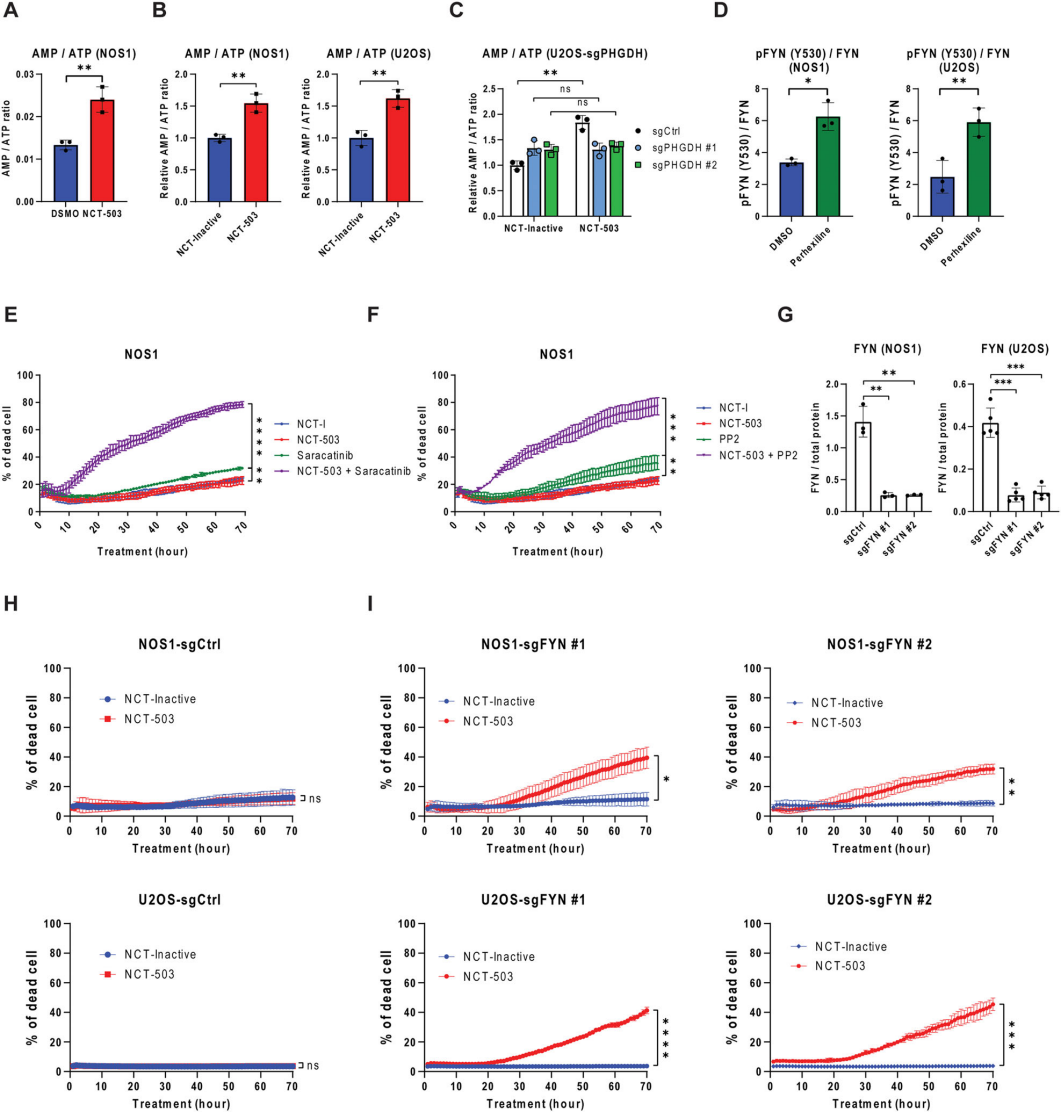
PHGDH inhibition elevates the AMP/ATP ratio, resulting in AMPKα activation, while perhexiline leads to AMPKα activation through FYN inhibition. **A** The AMP/ATP ratio of NOS1 cells quantified using intracellular metabolomics analysis. **B** The AMP/ATP ratio of OS cells measured using the colorimetric assay. **C** Quantification of AMP/ATP ratio of PHGDH KO U2OS cells under NCT-503 treatment. **D** The phosphorylation of FYN of OS cells under perhexiline treatment. Cell death induction of NOS1 cells under the combined inhibition of PHGDH and FYN with Src family kinase inhibitor saracatinib (**E**) or PP2 (**F**). **G** FYN expression of *FYN* KO OS cells. Cell death induction of control KO OS cells (**H**) and *FYN* KO OS cells (**I**) with NCT-503 treatment. All experiments are *n* = 3 at least. Bars represent means of values; error bars represent SEM. *P < 0.05, **P < 0.01, ***p < 0.005, ****p < 0.001.

NCT-503 treatment resulted in increased phosphorylation of AMPK at T172 in OS (Fig. 1I), and it was previously demonstrated that NCT-503 treatment leads to an increase in the AMP/ATP ratio [17]. To confirm the impact of NCT-503 on the AMP/ATP ratio in OS cells, the AMP/ATP ratio in OS cells treated with NCT-503 was quantified by both mass spectroscopy and colorimetric analysis. OS cells showed an increased AMP/ATP ratio under PHGDH inhibition with NCT-503 (Figs. 4A, B). To determine whether the elevated AMP/ATP ratio observed under NCT-503 treatment in OS cells is a result of on-target activity, PHGDH knockout (KO) was performed in U2OS cells (Fig. 4C and Supplementary Fig. 3A). PHGDH KO cells exhibited reduced proliferation and were less responsive to NCT-503 treatment compared to non-targeted control KO cells (Supplementary Fig. 3B). Additionally, PHGDH KO cells displayed increased sensitivity to single treatment of either perhexiline or ALPI3MT55 (Supplementary Fig. 3C, D). Furthermore, PHGDH KO U2OS cells demonstrated a significant induction of cell death compared to control cells (Supplementary Fig. 3C). Next, the AMP/ATP ratio increased in PHGDH KO cells compared to control KO when inhibited with NCT-503 (Fig. 4C). These results suggest that PHGDH inhibition with NCT-503 leads to an elevation in the AMP/ATP ratio, and this alteration subsequently triggers the activation of AMPK.

### Inhibition of FYN leads to increased OS cell death

FYN has been identified as an additional target of perhexiline; therefore, the phosphorylation status of FYN under perhexiline treatment was evaluated. Perhexiline treatment resulted in an increased inhibitory phosphorylation of FYN in OS cells (Fig. 4D). Next, synergy between FYN inhibition and PHGDH inhibition was determined. The FYN/Src family kinase inhibitors saracatinib and 4-amino-5-(4-chlorophenyl)-7-(t-butyl)pyrazolo[3,4-D]pyrimidine (PP2), were evaluated as single agents and in combination with NCT-503. Neither saracatinib nor PP2 induced effective cell death in OS cells as single agents. However, when combined with NCT-503, both saracatinib and PP2 FYN inhibitors led to a significant induction of cell death in OS cells (Fig. 4E, F). Furthermore, FYN knockout (KO) OS cells (Fig. 4G) were more sensitive to NCT-503 treatment and induction of cell death, while control KO cells did not show an induction of cell death with NCT-503 treatment (Fig. 4H, I).

### ALPI3MT55 induces apoptosis via FOXO activation and repression of the PI3 kinase/AKT pathway

**Fig. 5:**
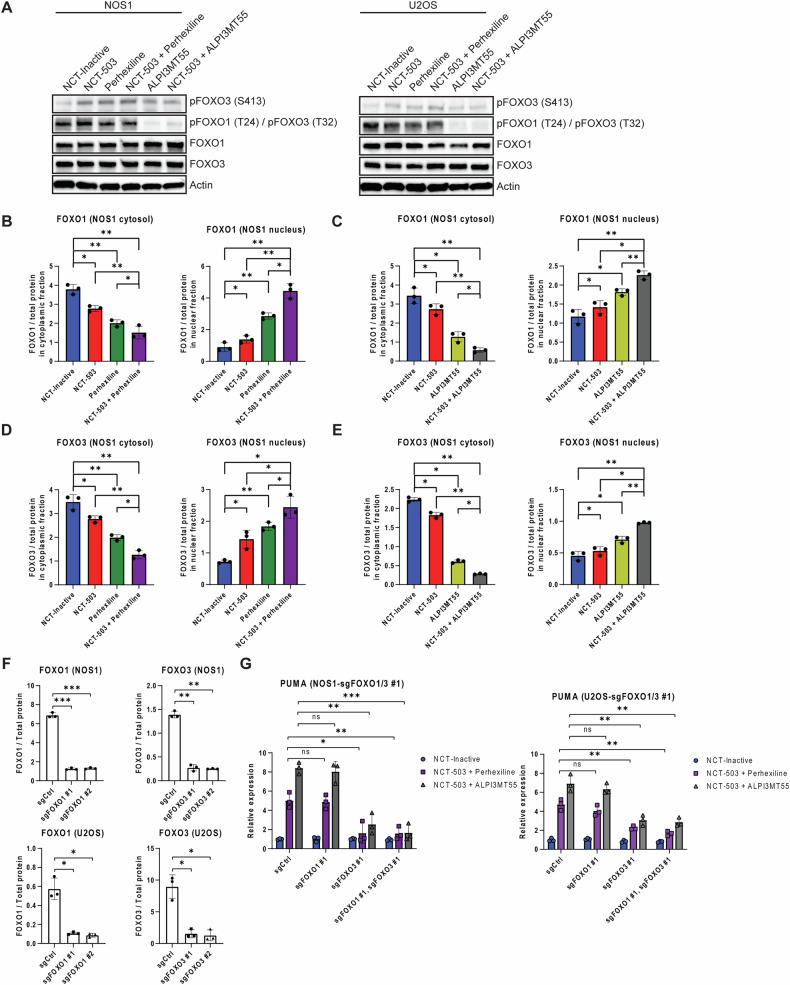
NCT-503, perhexiline, and ALPI3MT55 promote the nuclear localization of FOXO1/3, with FOXO3 being dominant in inducing cell death in OS cells under the combined treatments. **A** The phosphorylation of *FOXO* of OS cells under the combined treatments. Cytosolic/nuclear localization of FOXO1 protein of NOS1 cells under the combined treatment of NCT-503 and perhexiline (**B**) or ALPI3MT55 (**C**). Cytosolic/nuclear localization of FOXO3 protein of NOS1 cells under the combined treatment of NCT-503 and perhexiline (**D**) or ALPI3MT55 (**E**). **F** *FOXO1* and *FOXO3* knockouts in OS cells. **G** mRNA expression of *PUMA* in *FOXO1* KO, *FOXO3* KO, or *FOXO1/3* double KO cells under the combined treatments. All experiments are *n* = 3 at least. Bars represent means of values; error bars represent SEM. *P < 0.05, **P < 0.01, ***p < 0.005, ****p < 0.001.

The contribution of FOXO activation to apoptosis induction was assessed by determining the phosphorylation state of FOXO1 and FOXO3 under single and combination treatments. Both NCT-503 and perhexiline treatments in OS led to the activation of the FOXO pathway (Fig. 1D). Both NCT-503 and perhexiline single treatments increased phosphorylation at serine 413 of FOXO3, while their combined treatment further elevated this phosphorylation (Fig. 5A). Conversely, ALPI3MT55 treatment notably reduced inhibitory phosphorylation of FOXO1/3 at threonine 24/32 (Fig. 5A).

Next, the subcellular localization of FOXO3 was examined. Increased nuclear localization of FOXO1/3 was observed when cells were treated with either perhexiline or ALPI3MT55 (Fig. 5B, C). Combination treatment of NCT-503 and either perhexiline or ALPI3MT55 further enhanced this effect (Fig. 5D, E).

To understand whether FOXO1 or FOXO3 was more dominant in the induction of cell death caused by the combined treatment of NCT-503 and either perhexiline or ALPI3MT55, FOXO1 KO, FOXO3 KO, and FOXO1/3 double KO OS cells were generated (Fig. 5F). In FOXO1 KO cells, both combined treatments induced PUMA expression to the same level as in the control KO cells. However, in FOXO3 KO cells, the transcriptional induction of PUMA by either of the combined treatments was limited, and the mRNA expression level of PUMA in FOXO3 KO cells was similar to that in FOXO1/3 double KO cells (Fig. 5G and Supplementary Fig. 4A).

### Activation of FOXO3 represses c-MYC expression by inducing GSK3-dependent proteasomal degradation

Activation of FOXOs leads to the repression of c-MYC expression [35–37]. Therefore, the protein expression of c-MYC under the combined treatment of NCT-503 with either perhexiline or ALPI3MT55 was quantified. Each of the combination treatments significantly repressed the protein expression of c-MYC in OS cells (Supplementary Fig. 5A). In contrast, the transcription of the MYC gene was not altered in OS cells under these combined treatments (Supplementary Fig. 5B). When cycloheximide treatment was used with NCT-503 and either perhexiline or ALPI3MT55 in OS, each of the combined treatments promoted the destabilization of c-MYC protein in OS cells (Supplementary Fig. 5C). GSK3 phosphorylation mediates proteasomal targeting and degradation of MYC protein [37, 38]. To understand whether the promotion of MYC protein instability caused by the combination treatment of NCT-503 and perhexiline/ALPI3MT55 is dependent on proteasomal degradation, NOS1 cells were treated with GSK3 inhibitor BIO and 20S proteasome inhibitor Bortezomib [39, 40]. Both inhibitor treatments resulted in the recovery of c-MYC protein expression in NOS1 cells under the combined treatment of NCT-503 and either perhexiline or APLI3MT55 (Supplementary Fig. 5D). In addition, FOXO3 KO cells exhibited higher expression of c-MYC protein under each of the combined treatments compared to the control KO cells (Supplementary Fig. 5E). MYC-targeted cell cycle-related genes *CDK1*, *CCNA2*, and *CCNB1* were assessed in NOS1 cells under the combined treatments to determine the impact on OS cell proliferation. Each single treatment of NCT-503, perhexiline, and ALPI3MT55 repressed the transcription of these genes. Furthermore, the combined treatment of NCT-503, with either perhexiline or ALPI3MT55, resulted in a more pronounced inhibition of expression of these MYC target genes (Supplementary Fig. 5F). As FOXO3 is also known to inhibit mitochondrial gene expression by reducing c-MYC stability, the transcriptional expression of c-MYC target mitochondrial genes in NOS1 cells under the combination treatment of NCT-503 and perhexiline or ALPI3MT55 were quantified. Each single treatment of NCT-503, perhexiline, and ALPI3MT55 also repressed the transcription of *PPARGC1B*, *PPRC1*, *TFAM*, and *TOMM20*, and the combined treatment of NCT-503 and either perhexiline or ALPI3MT55 led to further repression of these gene’s transcription (Supplementary Fig. 5G) and protein expressions (Supplementary Fig. 5H).

### The triple combined treatment of NCT-503, perhexiline, and ALPI3MT55 significantly induced cell death in OS

**Fig. 6:**
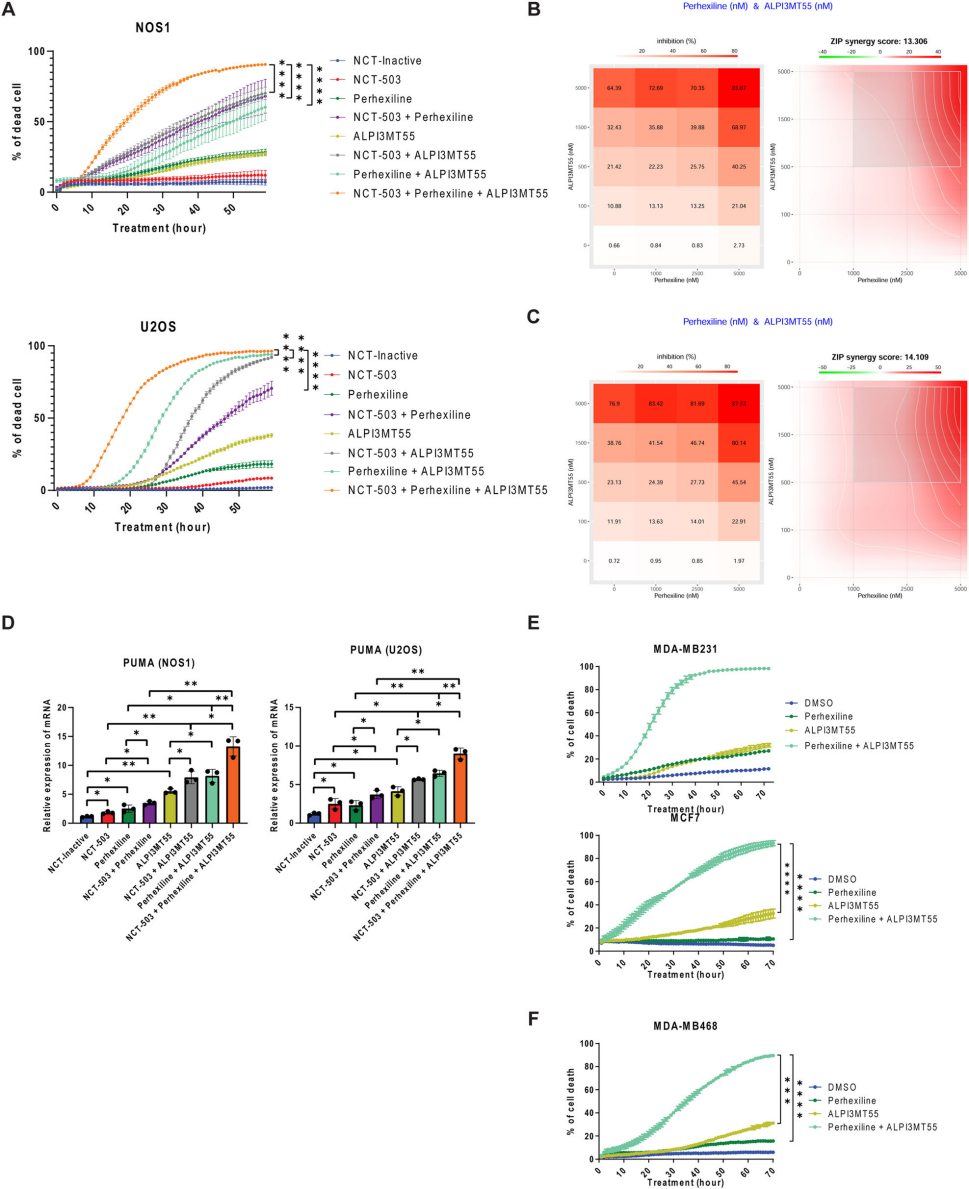
Triple combined treatment of NCT-503, perhexiline, and ALPI3MT55 significantly induce cell death in vitro. **A** Cell death induction of OS cells under triple combined treatment of NCT-503, perhexiline, and ALPI3MT55. The evaluation of synergy of combined treatment of perhexiline and ALPI3MT55 with NCT-503 (**B**) and without NCT-503 (**C**) using SynergyFinder interactive analysis. **D** Transcriptional activation of *PUMA* in OS cells under the treatment of NCT-503, perhexiline, and ALPI3MT55. Cell death induction of PHGDH low expression MDA-MB231 cells (**E**) and PHGDH high expression MDA-MB468 cells (**F**) under the combined treatment of perhexiline and ALPI3MT55. All experiments are *n* = 3 at least. Bars represent means of values; error bars represent SEM. *P < 0.05, **P < 0.01, ***p < 0.005, ****p < 0.001.

NCT-503, perhexiline, and ALPI3MT55 activate FOXO3 through distinct mechanisms, and the combination of NCT-503 with either perhexiline or ALPI3MT55 effectively induces apoptosis in OS cells. Therefore, the potential of the combined treatment of NCT-503, perhexiline, and ALPI3MT55 was evaluated by treating OS cells with perhexiline and ALPI3MT55, with or without NCT-503 (Fig. 6A). Of note, the combination of perhexiline and ALPI3MT55 significantly induced apoptotic cell death compared to each single treatment. Furthermore, the triple combined treatment resulted in even greater apoptosis in OS cells as seen by increased PARP cleavage and increased Annexin V staining (Fig. 6A, Supplementary Fig. 6D–F). On the other hand, none of these combinations were found to induced cell death in primary osteoblasts (Supplementary Fig. 6G). Additionally, the combined treatments led to a cell cycle arrest in the G1 phase (Supplementary Fig. 6H).

Next, the synergy between perhexiline and ALPI3MT55 in inducing cell death in OS cells was evaluated. Cell death induction was assessed at varying concentrations of these non-rapalog mTORC1 inhibitors, with or without NCT-503, using SynergyFinder (Fig. 6B, C). Perhexiline and ALPI3MT55 exhibited synergy both with each other and when combined with NCT-503 (Fig. 6A–C). The combination of perhexiline and ALPI3MT55 significantly increased PUMA mRNA expression, similar to the NCT-503 and ALPI3MT55 combination (Fig. 6A). Moreover, the triple combination of NCT-503, perhexiline, and ALPI3MT55 most significantly enhanced PUMA transcription compared to other treatments.

To explore the potential for inducing cell death independent of PHGDH inhibition, a combined treatment of perhexiline and ALPI3MT55 was tested on various cancer cell lines, including those with low and high PHGDH expression. This combination effectively induced cell death in multiple cell lines, such as PHGDH low-expression lines MDA-MB231 and MCF7, and the high-expression line MDA-MB468 (Fig. 6E, F). It was also effective in PHGDH high-expression cell lines of small cell lung cancer (NCI-H69, NCI-H69AR, DMS114), synovial sarcoma (FUJI), and Ewing’s sarcoma (LUPI) (Supplementary Fig. 6A–C).

### The triple combined treatment of NCT-503, perhexiline, and ALPI3MT55 significantly inhibits OS tumor growth in vivo

**Fig. 7:**
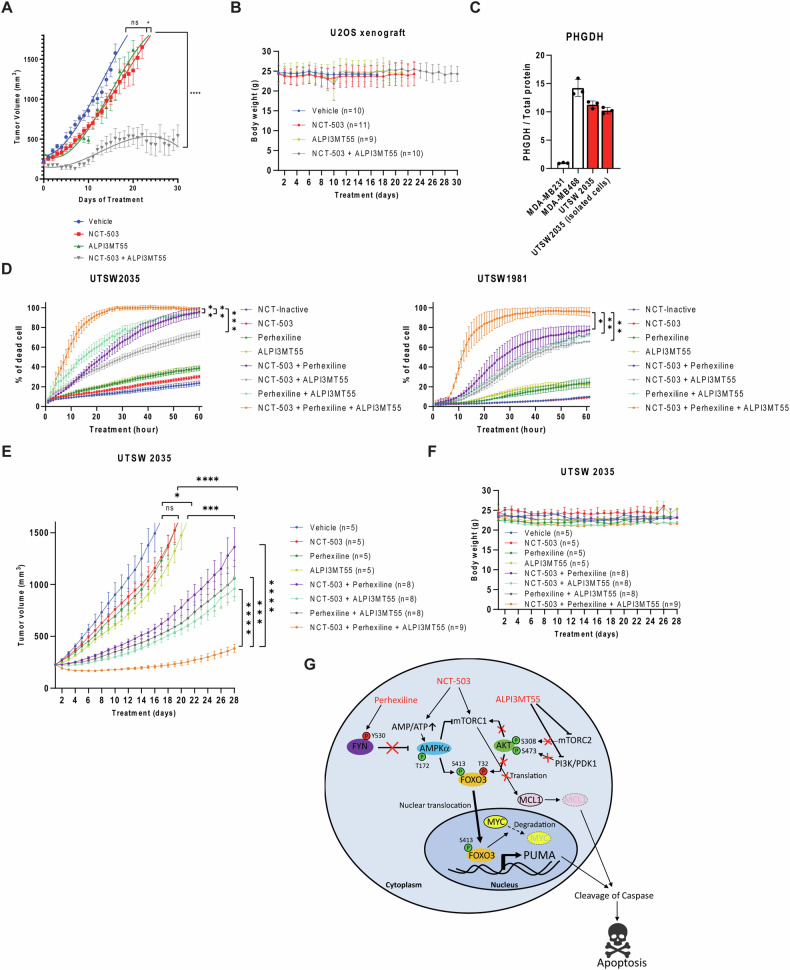
Triple combined treatment of NCT-503, perhexiline, and ALPI3MT55 in vivo. **A** Tumor growth curve of xenografted U2OS cells treated with NCT-503, ALPI3MT55, combination of NCT-503 and ALPI3MT55, or vehicle. **B** Body weight of xenografted mice with the treatment in (**A**). **C** PHGDH expression in UTSW2035 PDX and its isolated cells. **D** Cell death percentage of cells isolated from OS PDXs with the triple combined treatment of NCT-503, perhexiline, and ALPI3MT55 in vitro. **E** Tumor growth of the UTSW2035 PDX treated with NCT-503, perhexiline, and ALPI3MT55 in vivo. **F** Body weight of PDX tumor implanted mice. **G** Schematic model of apoptotic cell death induction by NCT-503, perhexiline, and ALPI3MT55. Bars represent means of values; error bars represent SEM. *P < 0.05, **P < 0.01, ***p < 0.00 5, ****p < 0.001.

To explore the effects of the combined treatment of NCT-503, perhexiline, and ALPI3MT55 in vivo, the efficacy of the combined treatment of NCT-503 and ALPI3MT55 was measured using an OS xenograft model with U2OS cells. The single treatment of NCT-503 or ALPI3MT55 did not result in the repression of OS tumor growth; however, the combined treatment of NCT-503 and ALPI3MT55 exhibited significant inhibition of OS tumor growth in vivo (Fig. 7A) without loss of body weight in the recipient mice (Fig. 7B). This is parallel to an experiment published for NCT-503 where the control arms are the same [7]. To evaluate the potential of triple combined treatment on OS, PHGDH high OS PDX models (Fig. 1A) were employed. Tumor cells were isolated from PHGDH high OS PDX tumors UTSW2035 and UTSW1981 (Fig. 7C) and cell death induction of the isolated cells under the triple combined treatment of NCT-503, perhexiline, and ALPI3MT55 was quantified in vitro. None of the single treatments with NCT-503, perhexiline, or ALPI3MT55 induced cell death; however, each of the double combined treatments induced cell death effectively, while the triple combined treatment induced cell death to the greatest extent (Fig. 7D). To confirm the result of the triple combined treatment on OS cells in vivo, the triple combined treatment was tested in vivo using an OS PDX mouse model (Fig. 7E). Single treatment of NCT-503, perhexiline, or ALPI3MT55 did not repress OS tumor growth, while each of the double combined treatments suppressed OS tumor growth. The triple combined treatment significantly enhanced tumor growth suppression over four weeks compared to each of the double combined treatments, without causing significant loss of body weight in the recipient mice (Fig. 7E, F). These results suggest a potential therapeutic strategy of combined treatment of NCT-503, perhexiline, and ALPI3MT55 for OS.

## Discussion

For over 40 years, OS has been treated with cisplatin, doxorubicin, and HD-MTX, yet progress in efficacy and reducing toxicity remains minimal. While the targeting of DHFR by methotrexate has long been standard of care, this treatment strategy requires in-patient hospitalization and is highly toxic. Replacement of this therapy by modern, less toxic, medications is warranted. The discovery that PHGDH expression in OS negatively correlates with survival revealed an avenue to explore in the quest to replace HD-MTX. Though the treatment for OS has not appreciably changed, our understanding of recurrent gene alterations is much better understood. In addition to the upregulation of PHGDH, several common gene alterations including *TP53*, *RB1*, *PTEN*, *MYC*, and *MDM2* that alter the DNA damage response, cell cycle, and PI3K-AKT-mTOR pathways have been identified [41–44]. Exploring these pathways’ intersections and dependencies may reveal how OS adapts to current treatments and offer new strategies to overcome treatment failure.

Given the importance of the folic acid cycle to OS, upstream enzymes to MTX were investigated as therapeutic targets. PHGDH emerged as a crucial enzyme in OS metabolism, with elevated expression linked to poor relapse-free and overall survival [7]. While PHGDH inhibition initially showed promise, it remained cytostatic due to compensatory mTORC1 pathway activation, limiting its effectiveness as a single agent therapy [17]. Consequently, combined inhibition of the mTORC1 pathway and inhibition of PHGDH was explored. The observation that the non-rapalog inhibitor of mTORC1, perhexiline, but not rapamycin itself, was effective in combination with PHGDH inhibition compelled further exploration of the mTORC pathway for a mechanistic explanation. The primary upstream signaling pathways for mTORC1, the AMPK and PI3K/Akt pathways, are intricately interconnected, and regulate mTORC1 activity and were therefore explored [45, 46].

mTORC1 regulation involves phosphorylation and dephosphorylation events within the AKT and AMPK pathways, modulating its activity. The PI3K/AKT/mTOR pathway is often hyperactivated in OS [47], while AMPK activation is linked to apoptotic cell death in OS [48–50]. Both pathways converge at FOXO1/3, key transcription factors in regulating apoptosis and metabolism [51]. GSEA results showed that OS cells treated with NCT-503 (PHGDH inhibitor) or perhexiline (AMPK activator) upregulated the FOXO pathway. This prompted us to use ALPI3MT55, a potent PI3K/AKT pathway inhibitor upstream of FOXO. The upregulation of the FOXO pathway by perhexiline also explains why non-rapalog mTORC inhibitors, but not rapamycin, lead to cell death when combined with NCT-503.

The proto-oncogene FYN, a member of the Src kinase family, is a non-receptor tyrosine kinase with numerous interacting molecules, including LKB1, a primary upstream kinase of AMPK. LKB1 phosphorylates and activates AMPK only when localized in the cytoplasm and becomes inactive in the nucleus [52, 53]. FYN phosphorylates LKB1 at tyrosine residues 261 and 365, restricting LKB1 to the nucleus and suppressing AMPK activation. Additionally, FYN phosphorylates PIKE-A at tyrosine residues 682 and 774, promoting its interaction with AMPK and further suppressing AMPK. Perhexiline treatment increases phosphorylation of tyrosine 531 in the Src Homology 2 (SH2) domain of FYN, inhibiting the interaction of FYN’s catalytic domain with its substrates. Further investigation into the precise mechanism by which perhexiline induces AMPK activation is warranted.

In addition to its role in AMPK activation, FYN regulates the AKT signaling pathway [54]. AKT, a serine/threonine kinase, plays crucial roles in apoptosis and proliferation and is activated via phosphorylation by several regulatory proteins. The interplay between AKT and FYN pathways is emerging as a key factor in invasive phenotypes in breast and pancreatic cancers. In pancreatic cancer cell lines, FYN mediates the AKT pathway via phosphorylation of the ionotropic glutamate receptor subunit 2B (GluN2B) at Ser1303, which subsequently phosphorylates AKT, enhancing tumor survival and progression [31, 55]. This phosphorylation mechanism also promotes lung cancer progression by enhancing ZNF322A protein stability. Given the regulatory role of FYN in cancer promotion by way of AKT, AKT inhibition presents a viable anti-tumor strategy. The synergy between perhexiline and ALPI3MT55, effective even without PHGDH inhibition, might be explained by the central role of FYN in regulating both the AKT and AMPK pathways.

The combined treatment of NCT-503, perhexiline, and ALPI3MT55 activates FOXO1 and FOXO3, leading to apoptosis, with FOXO3 being particularly dominant in OS cells. Activation of FOXO1 has been reported to inhibit OS oncogenesis by suppressing the Wnt/β-catenin pathway [56], and is targeted by miR-135b, which promotes OS cell proliferation and invasion by suppressing FOXO1 [57]. The combination treatment might inhibit OS tumor growth through these different mechanisms. In OS cells, the combination of perhexiline and ALPI3MT55, as well as NCT-503 and ALPI3MT55, demonstrated a pronounced transcriptional activation of PUMA compared to NCT-503 and perhexiline. ALPI3MT55 targets the PI3K/AKT pathway, while NCT-503 and perhexiline act on the AMPK pathway, activating FOXO3 through phosphorylation of different amino acids (Fig. 7G). This differential phosphorylation is crucial for interaction with 14-3-3 and subcellular localization of FOXO1/3. The combination of NCT-503 and perhexiline may be less effective in inducing PUMA due to their similar pathway targets. However, their distinct mechanisms for AMPK activation likely foster synergy. These mechanisms explain how the combination of NCT-503, perhexiline, and ALPI3MT55 synergistically induces cell death in OS cells.

This research has identified that metabolic adaptation to PHGDH inhibition involves AKT activation and AMPK alterations. Although the triple combination showed the greatest effect in vivo, developing a three-drug therapy is challenging as a PHGDH inhibitor has yet to enter phase I clinical trials. Meanwhile, the finding that the combination of ALPI3MT55 and perhexiline induces cell death presents as an immediate clinical opportunity. Combining ALPI3MT55 (can be made available from Advenchen), with perhexiline (repurposed from use in refractive angina), can proceed to dose finding in a phase I trial, aiming to eliminate the toxicity of HD-MTX.

## Materials and methods

### Plasmids and cell lines

LentiCRISPRv2-puro and lentiCRISPRv2-neo were purchased from Addgene. Sequences of sgRNAs are listed in Supplementary Materials. The human osteosarcoma NOS1 cells were obtained from RIKEN BioResource Research Center. The human osteosarcoma U2OS cells, human breast cancer MDA-MB231, MDA-MB468, and MCF7 cells, human small cell lung cancer NCI-H69, NCI-H69AR, and DMS114 cells, human embryonic kidney 293T cells were purchased from ATCC. Human synovial sarcoma Fuji cells were kindly provided from Dr. Kazuo Nagashima, Hokkaido University School of Medicine, Sapporo, Japan. Human Ewing sarcoma LUPI cells were a gift from John Pfeifer (Washington University in St Louis). Cells were cultured in DMEM (NOS1, U2OS, MDA-MB231, MDA-MB468, MCF7, and 293 T) or RPMI (NCI-H69, NCI-H69AR, DMS114, LUPI and Fuji) supplemented with 10% FBS (R&D systems), 1.3% pen/streptomycin (Gibco), and 1× plasmocin prophylactic (InvivoGen). All cells were checked for mycoplasma contamination regularly. CRISPR Knockout cell lines were established using lentiviruses produced in 293 T cells by co-expressing pCMV-deltaR8.2, pCMV-VSV-G, and LentiCRISPRv2 harvering sgRNA of interest. Cells were infected with lentiviruses for 18 h and subsequently subjected to selection using puromycin or Geneticin for a minimum of 1 week after infection.

### Automated cell imaging and quantitation

All automated cell imaging and quantification were done on cells in 96-well plates with Incucyte S3 (Sartorius) automated cell imaging and analysis system. For cell number counting, Cells were transduced nuclear-restricted red fluorescent protein mKate2 expression with Incucyte Nuclight Red Lentivirus (Sartorius) and selected with puromycin for stable expression. For enumerating cell number of OS PDX derived cells, cell-by-cell label-free adherent cell counts method was employed. The cell impermeable DNA-binding dye YOYO-1 Iodide (Thermo Fisher) was used to stain dead cells and Incucyte Caspase-3/7 Green Dye (Sartorius) and Incucyte Annexin V Dye (Sartorius) were used for detection of apoptotic cell death. For the quantification of cell cycle phase, U2OS cells were infected with Incucyte Cell Cycle Lentivirus Reagents (Sartorius) and selected with puromycin for stable expression. Incucyte software was used to count the red or green nuclei of transduced cells to measure the number of cells at each timepoint.

### NanoString metabolism gene panel and gene set enrichment analysis

NOS1 and Saos2 cells were treated with either 10 mM NCT-inactive, 10 mM NCT-inactive with 15 mM NCT-503, DMSO or 5 mM perhexiline for 24 h. RNA was collected using the Direct-zol RNA Miniprep Plus Kit (Zymo Research) and sent to NanoString. GSEA (https://www.gsea-msigdb.org) were performed on Partek Flow (Partek) using the KEGG database (https://www.kegg.jp/kegg/).

### Western blot

Cells were lysed in Cell Lysis Buffer (Cell Signaling) supplemented with protease and phosphatase inhibitors (Thermo Fisher). Protein concentration was measured using Protein Assay dye reagent (Bio-Rad) on Infinite M200 plate reader (Tecan). Protein expression was detected and quantified by Wes automated western blotting system (ProteinSimple) with 12-230 kDa separation module. For the detection of proteins that non valid antibodies were available for in WES system, BioRad mini-PROTEAN TGX gel system or Invitrogen NuPAGE Bis-Tris gel system with ChemiDoc XRS+ chemiluminescence detection system were used. Antibodies are listed in Supplementary information. Full western blots are available in Supplementary Materials and Methods.

### Nuclear/cytosolic fractionation

8 × 10^6^ cells NOS1 or 2 × 10^6^ cells of U2OS cells were seeded in 10 cm dishes and cultured for 24 h and then treated with indicated combination of inhibitors for 24 h. Nuclear/cytosolic fractionation was performed using NE-PER Nuclear and Cytoplasmic extraction reagents (Thermo Fisher) by following the manufacture protocol. Protein expression in nuclear and cytosolic fractions were detected and quantified using Wes automated western blotting system.

### Quantitative RT-PCR

Total RNA was collected using Direct-zol RNA Miniprep Plus kit (Zymo Research). RT-PCR was conducted using Superscript II Reverse Transcriptase (Invitrogen) with Oligo dT primer (Invitrogen) and Random Decamers (Ambion). The real-time PCR analyses using Power SYBR Green Master Mix (Thermo fisher) were performed on a CFX96 Touch Real-Time PCR Detection System (Bio-Rad) as previously described [58]. Sequences of oligonucleotides are listed in the Supplementary Materials and Methods.

### AMP/ATP ratio measurement

8 × 10^6^ cells NOS1 or 2 × 10^6^ cells of U2OS cells were plated in 10 cm dishes and cultured for 24 h and then treated with either 15uM of NCT-503 or Inactive control for 24 h. Number of cells were counted using Countess II automated cell counter (Invitrogen). Measurement of AMP and ATP were performed on Infinite M200 plate reader (Tecan) using AMP Colorimetric Assay Kit (BioBision) and ATP Colorimetric/Fluorometric Assay Kit (BioVision) respectively by following the manufacture protocols.

### The prediction of synergy of combined treatment

SynergyFinder interactive analysis 1.5 × 10^4^ cells NOS1 or 4 × 10^3^ cells of U2OS cells were seeded in 96well plate and cultured for 24 h, and were treated by combining various concentrations of perhexiline (0, 1, 2.5, or 5 μM) and ALPI3MT55 (0, 0.1, 0.5, 1.5, or 5 μM) with NCT-inactive (15μM) or NCT-503 (15 μM), and measured the induction of cell death under various combination using YOYO-1 Iodide dye and Incucyte S3 automated imaging system. The potential synergistic effects of combination treatment of perhexiline and ALPI3MT55 were predicted using SynergyFinder web-application (https://synergyfinder.fimm.fi/).

### Animal studies

All animal studies protocols were approved by Washington University in St. Louis Institutional Animal Care and Use Committee (IACUC). Mice were maintained under IACUC guidelines. Xenograft were performed as previously described [17]. Briefly 2 × 10^6^ U2OS cells were grafted by subcutaneous injection into the right flank of athymic nude mice (female, 4–6 weeks old, Jackson Laboratory). Mice were randomly assigned to treatment groups as xenograft derived tumors reached 200 mm^3^. Mice were administered vehicle (5% ethanol, 35% polyethylene glycol 300, 60% aqueous 30% hydroxyropyl-betacyclodextrin) or 40 mg/kg NCT-503 (in vehicle) intraperitoneally daily, and vehicle (water with few drops of tween-80) or 12 mg/kg ALPI3MT55 (in vehicle) by oral gavage daily.

OS PDX tumors were obtained from Peter Houghton, PhD, at The University of Texas Health Science Center. OS tumor pieces were cut into 2 × 2 mm fragments and implanted into right frank of NSG mice (female, 4-6 weeks old, Jackson Laboratory). Mice were randomly assigned to treatment groups as xenograft derived tumors reached 200 mm^3^. Mice were administered vehicle or 40 mg/kg NCT-503 intraperitoneally daily, and vehicle or 12 mg/kg ALPI3MT55 by oral gavage daily, and vehicle (1.8% w/v hydroxypropyl-beta-cyclodextrin) or 8 mg/kg perhexiline (in vehicle) by oral gavage daily. ALPI3MT55 (or its vehicle) and Perhexiline (or its vehicle) were administrated with a minimum eight-hour interval between them. Tumors were measured in two dimensions with a digital caliper daily and tumor volumes were calculated using the formula (length x width2)/2. Body weights were obtained prior to dosing each day and the dose of the compound was adjusted accordingly. Mice were euthanized after 30 days of treatment (xenograft), 4weeks of treatment (PDX) or when tumors reached 2000 mm^3^.

### Generation of PDX cell lines

The OS PDX tumor tissue was minced using a surgical blade and incubated with dissociation buffer containing 2 mg/ml collagenase (Sigma), 0.005% Trypsin (Gibco) in DMEM, while gently agitating for 90 minutes at 37 °C. After the incubation, the cells were collected by centrifugation and treated with a red blood cell lysis buffer (Sigma) to remove any remaining red blood cells. To enrich the human OS cells from the mixed cell population in isolated cells from PDX tumor, the mouse derived cell removal was performed using a mouse cell depletion kit (Miltenyi biotec). OS PDX derived cells were cultured in DMEM supplemented with 10%FBS, 1.3% pen/streptomycin, and 1× plasmocin prophylactic.

### Isolation of mouse primary osteoblasts

Primary osteoblasts were isolated from 1- to 3-day-old neonatal C57bl/6 mice. Calvariae were excised using scissors, washed with PBS, and carefully cleaned to remove all associated connective tissue and brain matter using fine forceps. The calvariae were then bisected and placed in PBS, followed by shaking at 37 ° C for 10 min. The calvariae were enzymatically digested in 10 ml of 200 U/ml collagenase (Worthington Biochemical Corp) with constant shaking at 37 °C for 10 min. The first digestion fraction was discarded. The calvariae were then subjected to a second digestion with 10 ml of 200 U/ml collagenase, shaken at 37 °C for 15 min, with this fraction also discarded. For the third digestion, the calvariae were treated with 10 ml of 200 U/ml collagenase and shaken at 37 °C for 15 min. This third digestion fraction was collected. The digestion was repeated two more times, and the fourth and fifth fractions were combined with the third fraction. The combined fractions were filtered through a 70 μM cell strainer and centrifuged at 1500 rpm for 5 minutes. The resulting cell pellet was resuspended in αMEM (Gibco) supplemented with 10% FBS and 1% penicillin/streptomycin, and then seeded in 100 mm tissue culture plates. The cells were cultured in αMEM supplemented with 10% FBS and 1% penicillin/streptomycin.

### Quantification and statistical analysis

Statistical tests were performed in GraphPad Prism 10 software. Differences between time series were analyzed by 2-way ANOVA. Grouped data were analyzed by unpaired t test. All error bars show standard deviation. *P*-values are denoted in the following way: ns: *p* > 0.05; *: *p* ≤ 0.05; **: *p* ≤ 0.01; ***: *p* ≤ 0.005; ****: *p* ≤ 0.001.

## Supplementary information

Supplementary information

Supplemental table 1

Western raw data

## Data Availability

The datasets generated and/or analyzed during the current study are available from the corresponding author on reasonable request. All data from this study are provided within the article and its supplementary information files.
