# Supplementary material for: PHGDH inhibition and FOXO3 modulation drives PUMA-dependent apoptosis in osteosarcoma
Source: Cell Death Dis. 2025 Feb 12;16(1):89. doi: 10.1038/s41419-025-07378-6 (PMC11814296; doi:10.1038/s41419-025-07378-6)
Supplement: Supplementary file 1 — Supplementary information [file 41419_2025_7378_MOESM1_ESM.pdf]

A

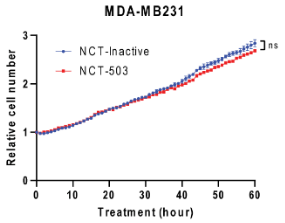

B

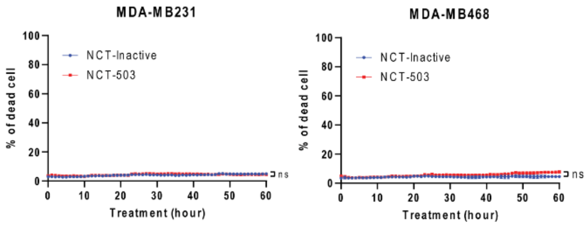

C

Enriched pathways under NCT-503 treatment

Relaxin signaling pathway
AGE-RAGE signaling pathway in diabetic complications
Human T-cell leukemia virus 1 infection
Alanine, aspartate and glutamate metabolism
Hepatocellular carcinoma
Melanoma
Human cytomegalovirus infection
Fc epsilon RI signaling pathway
Growth hormone synthesis, secretion and action
Endocytosis
Estrogen signaling pathway
C-type lectin receptor signaling pathway
MAPK signaling pathway
ErbB signaling pathway
Oxytocin signaling pathway

Enriched pathways under perhexiline treatment

Thermogenesis
AMPK signaling pathway
Oxidative phosphorylation
Th17 cell differentiation
Glycolysis / Gluconeogenesis
Insulin resistance
Non-alcoholic fatty liver disease (NAFLD)
Small cell lung cancer
Central carbon metabolism in cancer
Purine metabolism
Pathways in cancer
Retrograde endocannabinoid signaling
Huntington disease
Human immunodeficiency virus 1 infection
Hematopoietic cell lineage
HIF-1 signaling pathway
Signaling pathways regulating pluripotency of stem cells
Autophagy – animal
Parkinson disease
Proteoglycans in cancer
Metabolic pathways
Th1 and Th2 cell differentiation
Osteoclast differentiation

D

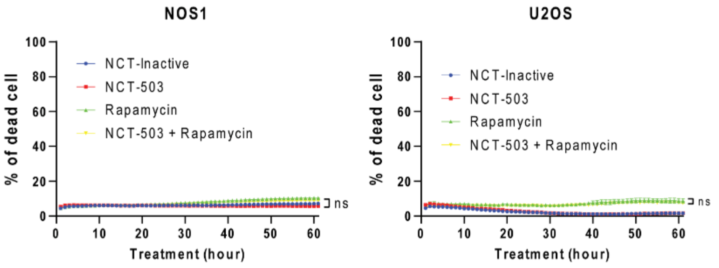

E

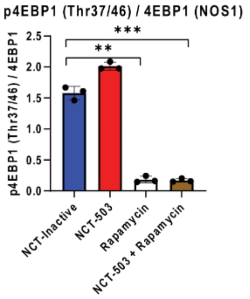

Supplementary Fig. 1 PHGDH inhibition attenuated cell proliferation without causing cell death in OS. A, B NCT-503 treatment on PHGDH-low expressing MDA-MB231 cells and PHGDH-high expressing MDA-MB468 cells. PHGDH inhibition effectively repressed cell proliferation of MDA-MB468 but not MDA-MB231 cells. **C** GSEA on NOS1 cells treated with NCT-503 or perhexiline treatment. **D** Rapamycin did not induce significant cell death when combined with NCT-503 in OS cells. **E** Rapamycin suppresses mTORC1 activity even when combined with NCT-503. All experiments are n=3 at least. Bars represent means of values; error bars represent SEM. *P<0.05, **P<0.01, ***p<0.005, ****p<0.001.

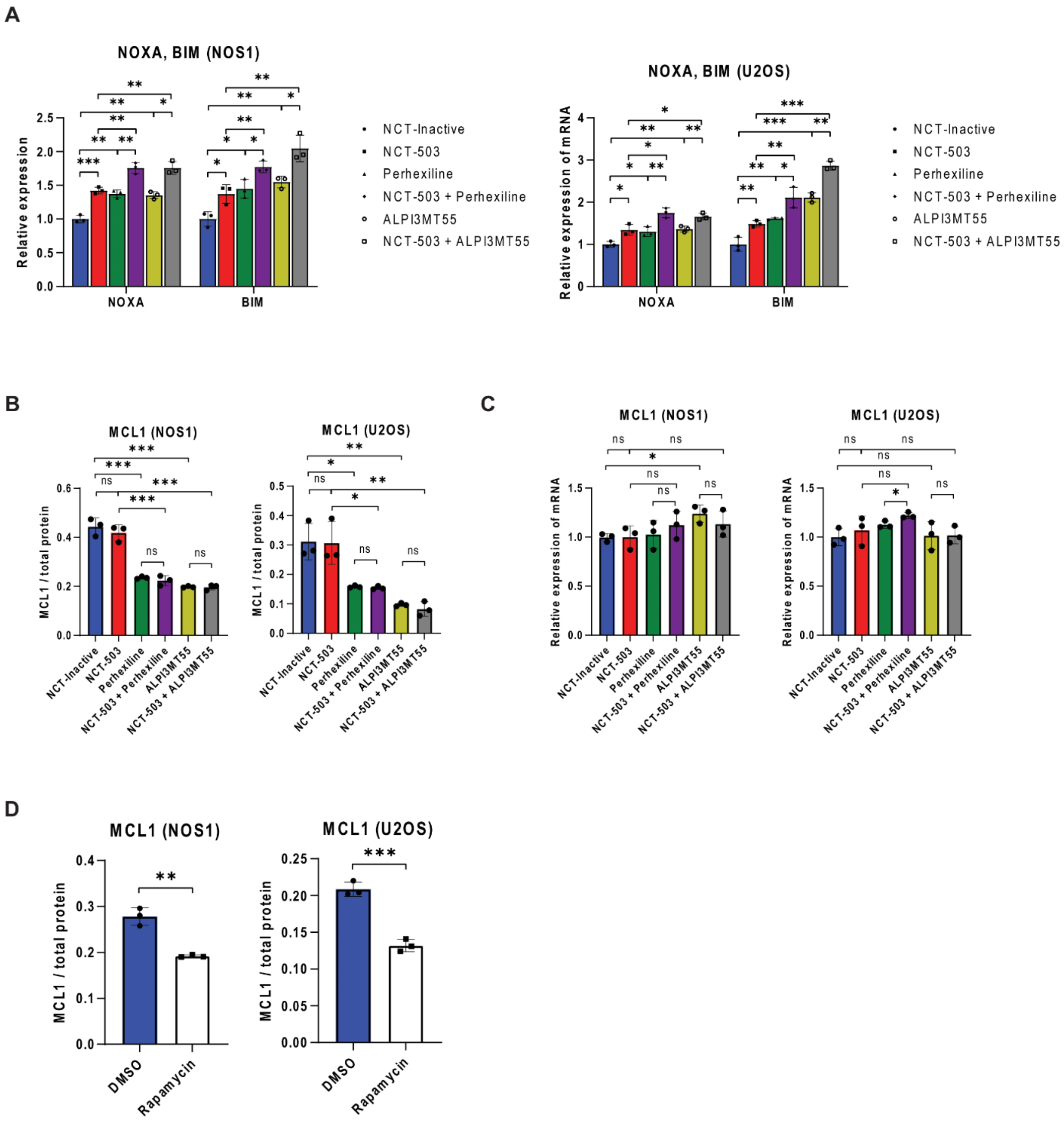
A
NOXA, BIM (NOS1)
Relative expression
NOXA
BIM
NOXA, BIM (U2OS)
Relative expression of mRNA
NCT-Inactive
NCT-503
Perhexiline
NCT-503 + Perhexiline
ALPI3MT55
NCT-503 + ALPI3MT55
B
MCL1 (NOS1)
MCL1 / total protein
MCL1 (U2OS)
C
MCL1 (NOS1)
Relative expression of mRNA
MCL1 (U2OS)
D
MCL1 (NOS1)
MCL1 / total protein
DMSO
Rapamycin
MCL1 (U2OS)

Supplementary Fig. 2 The combined treatment of NCT-503 with perhexiline or ALPI3MT55 promoted transcription of the pro-apoptotic genes *NOXA* and *BIM*. A mRNA expression of *NOXA* and *BIM* in OS cells under the combined treatments. **B, C** Protein expression (B) and mRNA (C) of MCL1 in OS cells under the combined treatments. **D** MCL1 protein expression in OS cells with rapamycin treatment. All experiments are n=3 at least. Bars represent means of values; error bars represent SEM. *P<0.05, **P<0.01, ***p<0.005, ****p<0.001.

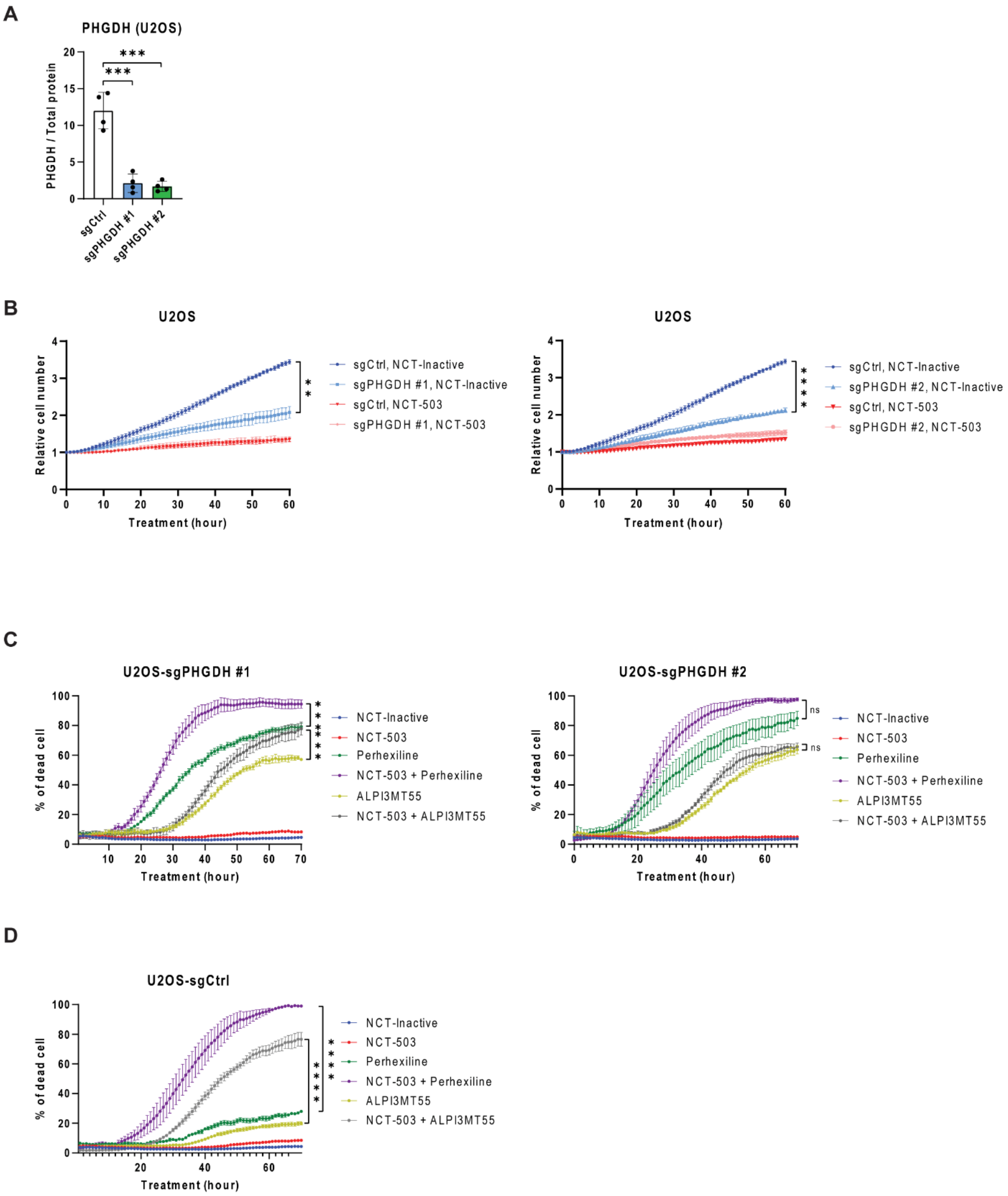
A
PHGDH (U2OS)
PHGDH / Total protein
***
***
sgCtrl
sgPHGDH #1
sgPHGDH #2
B
U2OS
Relative cell number
Treatment (hour)
sgCtrl, NCT-Inactive
sgPHGDH #1, NCT-Inactive
sgCtrl, NCT-503
sgPHGDH #1, NCT-503
U2OS
sgCtrl, NCT-Inactive
sgPHGDH #2, NCT-Inactive
sgCtrl, NCT-503
sgPHGDH #2, NCT-503
C
U2OS-sgPHGDH #1
% of dead cell
Treatment (hour)
NCT-Inactive
NCT-503
Perhexiline
NCT-503 + Perhexiline
ALPI3MT55
NCT-503 + ALPI3MT55
U2OS-sgPHGDH #2
ns
ns
D
U2OS-sgCtrl
% of dead cell
Treatment (hour)
NCT-Inactive
NCT-503
Perhexiline
NCT-503 + Perhexiline
ALPI3MT55
NCT-503 + ALPI3MT55

Supplementary Fig. 3 Knockout of PHGDH sensitizes OS cells to perhexiline or ALPI3MT55 treatment, leading to the induction of cell death. A PHGDH expression in *PHGDH* KO U2OS cells. **B** Measurement of cell proliferation of *PHGDH* KO U2OS cells treated with NCT-503. **C, D** Percentage of cell death of *PHGDH* KO U2OS cells (C) and control KO U2OS cells (D) under combined treatment of NCT-503 and perhexiline or ALPI3MT55. All experiments are n=3 at least. Bars represent means of values; error bars represent SEM. *P<0.05, **P<0.01, ***p<0.005, ****p<0.001.

A

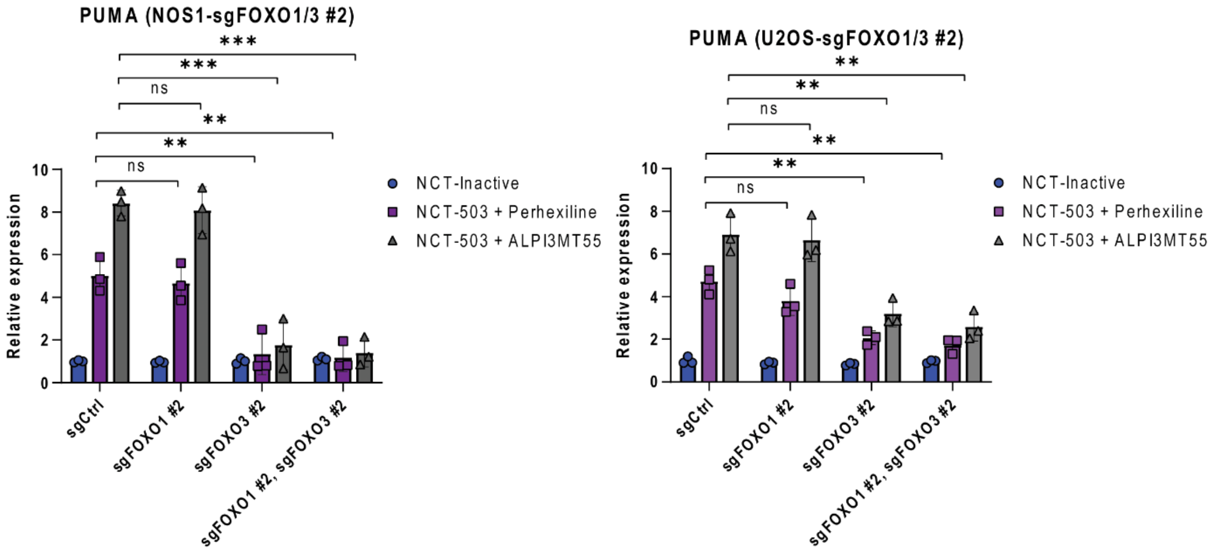

Supplementary Fig. 4 FOXO3 is critical for the induction of PUMA transcription in OS cells under the combined treatments. A mRNA expression of *PUMA* in *FOXO1* KO, *FOXO3* KO, and *FOXO1/3* double KO cells. All experiments are n=3 at least. Bars represent means of values; error bars represent SEM. *P<0.05, **P<0.01, ***p<0.005, ****p<0.001.

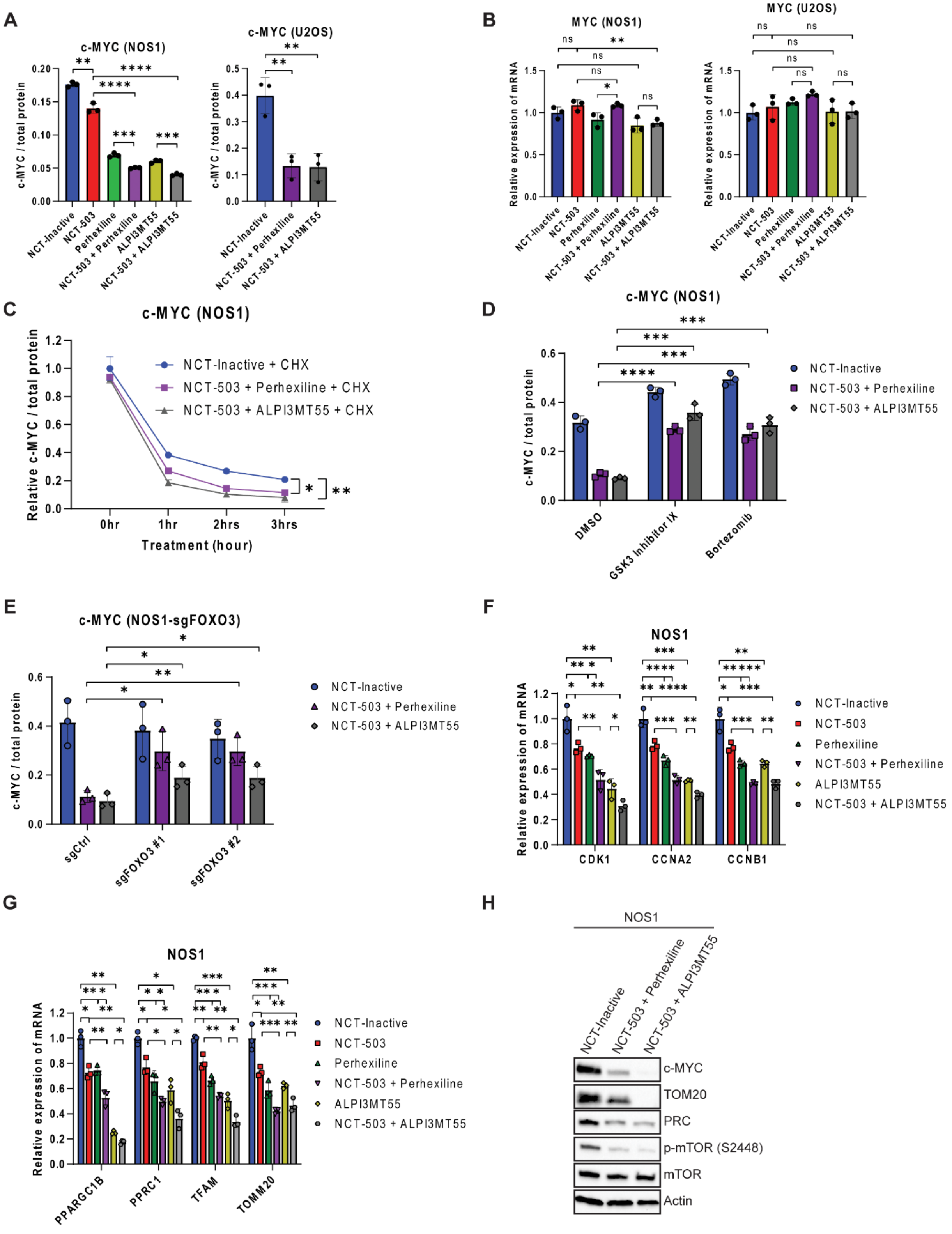
A
c-MYC (NOS1)
c-MYC / total protein
NCT-Inactive
NCT-503
Perhexiline
NCT-503 + Perhexiline
ALPI3MT55
NCT-503 + ALPI3MT55
c-MYC (U2OS)
NCT-503 + Perhexiline
NCT-503 + ALPI3MT55
B
MYC (NOS1)
MYC (U2OS)
Relative expression of mRNA
ns
C
c-MYC (NOS1)
Relative c-MYC / total protein
NCT-Inactive + CHX
NCT-503 + Perhexiline + CHX
NCT-503 + ALPI3MT55 + CHX
0hr
1hr
2hrs
3hrs
Treatment (hour)
D
c-MYC (NOS1)
c-MYC / total protein
DMSO
GSK3 Inhibitor IX
Bortezomib
NCT-Inactive
NCT-503 + Perhexiline
NCT-503 + ALPI3MT55
E
c-MYC (NOS1-sgFOXO3)
sgCtrl
sgFOXO3 #1
sgFOXO3 #2
F
NOS1
CDK1
CCNA2
CCNB1
G
NOS1
PPARGC1B
PPRC1
TFAM
TOMM20
H
NOS1
c-MYC
TOM20
PRC
p-mTOR (S2448)
mTOR
Actin

Supplementary Fig. 5 Activation of FOXO3 results in c-MYC degradation in osteosarcoma. A, B Protein (A) and mRNA (B) expression of c-MYC of OS cells under the combined treatments. **C** Time course curve of c-MYC protein expression in NOS1 cells under the combined treatments with CHX. **D** c-MYC protein expression in NOS1 cells under the combined treatments with GSK3 inhibitor IX or proteasome inhibitor bortezomib. The expression levels of c-MYC at each time point for each treatment were normalized against the c-MYC expression at 0 hours in the control treatment. **E** The protein expression of c-MYC in *FOXO3* KO NOS1 cells under the combined treatments. **F** mRNA expression of cell cycle related genes in NOS1 cells under the combined treatments. **G** mRNA expression of mitochondria related genes in NOS1 cells under the combined treatments. **H** The expression of mitochondria related proteins under the combined treatments. All experiments are n=3 at least. Bars represent means of values; error bars represent SEM. *P<0.05, **P<0.01, ***p<0.005, ****p<0.001.

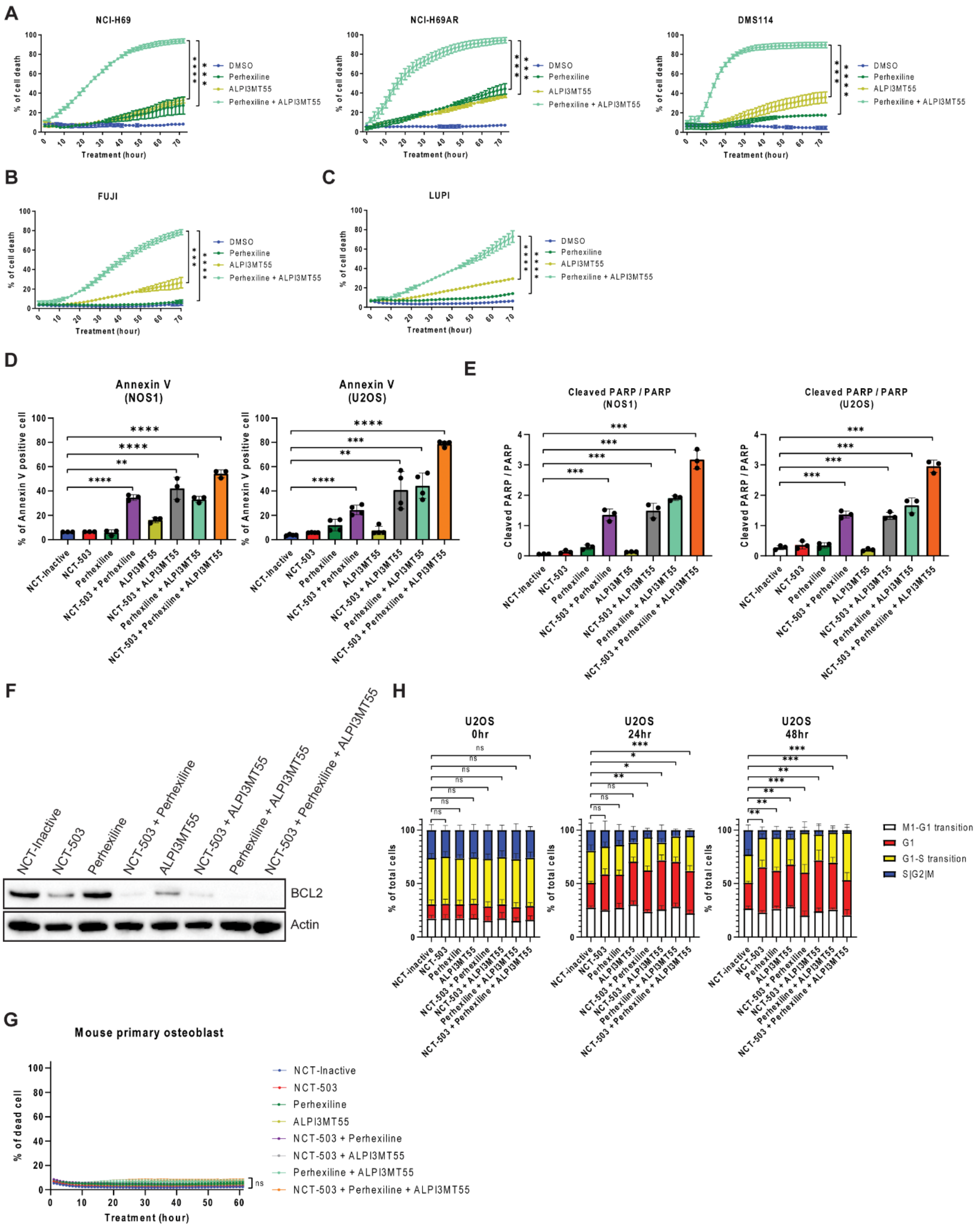
A
NCI-H69
NCI-H69AR
DMS114
% of cell death
Treatment (hour)
DMSO
Perhexiline
ALPI3MT55
Perhexiline + ALPI3MT55
B
FUJI
C
LUPI
D
Annexin V (NOS1)
Annexin V (U2OS)
% of Annexin V positive cell
E
Cleaved PARP / PARP (NOS1)
Cleaved PARP / PARP (U2OS)
NCT-Inactive
NCT-503
Perhexiline
NCT-503 + Perhexiline
ALPI3MT55
NCT-503 + ALPI3MT55
Perhexiline + ALPI3MT55
NCT-503 + Perhexiline + ALPI3MT55
F
BCL2
Actin
H
U2OS 0hr
U2OS 24hr
U2OS 48hr
% of total cells
M1-G1 transition
G1
G1-S transition
S|G2|M
G
Mouse primary osteoblast
% of dead cell
Treatment (hour)
ns

Supplementary Fig. 6 Combined treatment of perhexiline and ALPI3MT55 induces cell death in different types of cancer cells. A, B, C Combined treatment of perhexiline and ALPI3MT55 in lung cancer cell lines (A), Synovial sarcoma (B), and Ewing's sarcoma (C). **D** Percentage of annexin V positive cells under the combined treatments at 24 hours **E, F** Expression of cleaved PARP (E) and Bcl2 (F) under the combined treatments in OS cells. **G** Cell death induction in mouse osteoblast cells under triple combined treatment of NCT-503, perhexiline, and ALPI3MT55. **H** Percentage of cell cycle phase distribution of U2OS cells at 0, 24, and 48hr under the combined treatment. Statistical analysis was performed for the percentage of cells in the G1 and G1-S transition phases under each treatment. All experiments are n=3 at least. Bars represent means of values; error bars represent SEM. *P<0.05, **P<0.01, ***p<0.005, ****p<0.001.

SUPPLEMENTARY MATERIALS AND METHODS

Plasmids

lentiCRISPRv2 puro (addgene #98290)

lentiCRISPRv2 neo (addgene #98292)

pCMV delta R8.2 (addgene #12263)

pCMV-VSV-G (addgene #8454)

lentiCRISPR v2 puro-sgCtrl

lentiCRISPR v2 puro-sgPHGDH #1

lentiCRISPR v2 puro-sgPHGDH #2

lentiCRISPR v2 puro-sgBBC3 (PUMA) #1

lentiCRISPR v2 puro-sgBBC3 (PUMA) #2

lentiCRISPR v2 puro-sgFOXO3 #1

lentiCRISPR v2 puro-sgFOXO3 #2

lentiCRISPR v2 neo-sgCtrl

lentiCRISPR v2 neo-sgPHGDH #1

lentiCRISPR v2 neo-sgPHGDH #2

lentiCRISPR v2 neo-sgBBC3 (PUMA) #1

lentiCRISPR v2 neo-sgBBC3 (PUMA) #2

lentiCRISPR v2 neo-sgFOXO1 #1

lentiCRISPR v2 neo-sgFOXO1 #2

lentiCRISPR v2 neo-sgFYN #1

lentiCRISPR v2 neo-sgFYN #2

Chemicals

NCT-503 (Sigma SML1659), NCT-503 (Selleck S8619)

NCT-503 inactive control (Sigma SML1671)

Perhexiline (Sigma SML0120)

ALPI3MT55 (Advenchen Laboratories)

Rapamycin (Sigma R8781)

Z-VAD- FMK (Enzo BML-P416-001)

GSK-3 Inhibitor IX BIO (Sigma 361550)

Bortezomib (Cayman chemical 10008822)

Saracatinib (Cayman chemical 11497)

PP2 (Cayman chemical 13198)

YOYO-1 (ThermoFisher Y3601)

Caspase-3/7 Dye for Apoptosis (Sartorius 4440)

Lipofectamine 2000 (Invitrogen 11668027)

Hexadimethrine bromide (Polybrene) (Sigma H9268)

Antibodies

anti-PHGDH antibody (Sigma HPA021241)

anti-Caspase3 antibody (Novus NB100-56708)

anti-PUMA antibody (Cell signaling 4976)

anti-4E-BP1 antibody (Cell signaling 9452)

anti-phospho-4E-BP1 (Thr37/46) antibody (Cell signaling 2855)

anti-AKT antibody (Cell signaling 4691)

anti-phospho-AKT (S308) antibody (Cell signaling 13038)

anti-phospho-AKT (Ser 473) antibody (Cell signaling 4060)

anti-AMPKα antibody (Cell signaling 5831)

anti-phospho-AMPKα (Thr172) (Cell signaling 2535)

anti-FoxO1a antibody (Cell signaling 2880)

anti-FoxO3a antibody (Cell signaling 2497)

anti-phospho-FoxO3a (Ser 413) antibody (Cell signaling 8174)

anti-phospho-FoxO1 (Thr24)/FoxO3a (Thr32) antibody (Cell signaling 9464)

anti-FYN antibody (Abcam ab182661)

anti-phospho-FYN (Y530) antibody (Abcam ab182661)

anti-PRC (Santa Cruz sc-376431)

anti-Tomm20 (Santa Cruz sc-17764)

anti-mTOR (Cell signaling 2972)

anti-phospho-mTOR (Ser 2448) (Cell signaling 2971)

anti-GAPDH antibody (Novus NB300-328)

anti-c-MYC antibody (Abcam ab32072)

BCL-2 antibody (Millipore Sigma SAB4500003)

PARP antibody (Cell signaling 9542)

Cleaved PARP (Cell signaling 5625)

anti-β-Actin antibody (Sigma a1978).

Oligos

sgRNAs for gene knockout

sgPHGDH #1	AGCTGCGTTGATGACATCAG
sgPHGDH #2	CATTCCACAAGTGAGTTCTG
sgBBC3 #1	TCAACGCACAGTACGAGCGG
sgBBC3 #2	CTCCTCCCCGCGGACTCCCG
sgFOXO1 #1	CAAGGCCATCGAGAGCTCGG
sgFOXO1 #2	GAGTTGGACTGGCTAAACTC
sgFOXO3 #1	CGACTCCATGATCCCCGAGG
sgFOXO3 #2	CTGGGGAAACCTGTCCTACG
sgFYN #1	TGAACTCTTCGTCTCATACG
sgFYN #2	CAACAACTTCCACGCAGCCG
sgControl	TGCGAATACGCCCACGCGAT

Primers for real-time PCR

PUMA (BBC3) F	ACGACCTCAACGCACAGTACG
PUMA (BBC3) R	GTAAGGGCAGGAGTCCCATGATG
NOXA (PMAIP1) F	GGAGATGCCTGGGAAGAAGG
NOXA (PMAIP1) R	TCCTGAGCAGAAGAGTTTGG
Bim (BCL2L11) F	CCAGCACCCATGAGTTGTGACAA
Bim (BCL2L11) R	GCGTTAAACTCGTCTCCAATACGCC
c-Myc (MYC) F	GTCCTCGGATTCTCTGCTCTCC
c-Myc (MYC) R	GCTGTGAGGAGGTTTGCTGTG
MCL1 F	GCTGCATCGAACCATTAGCAGAAAG
MCL1 R	TTGGAGTCCAACTGCATAAACTGGT

PGC1β (PPARGC1B) F	GATGCCAGCGACTTTGACTC
PGC1β (PPARGC1B) R	ACCCACGTCATCTTCAGGGA
PRC (PPRC1) F	CAAGCGCCGTATGGGACTTT
PRC (PPRC1) R	GGAGGCATCCATGTAGCTCT
TFAM F	AGCTCAGAACCCAGATGC
TFAM R	CCACTCCGCCCTATAAGC
TOMM20 F	GGTACTGCATCTACTTCGACCG
TOMM20 R	TGGTCTACGCCCTTCTCATATTC
CDK1 F	AAACTACAGGTCAAGTGGTAGCC
CDK1 R	TCCTGCATAAGCACATCCTGA
Cyclin A2 (CCNA2) F	GGATGGTAGTTTTGAGTCACCAC
Cyclin A2 (CCNA2) R	CACGAGGATAGCTCTCATACTGT
Cyclin B1 (CCNB1) F	AACTTTCGCCTGAGCCTATTTT
Cyclin B1 (CCNB1) R	TTGGTCTGACTGCTTGCTCTT
MCL1 F	GCTGCATCGAACCATTAGCAGAAAG
MCL1 R	TTGGAGTCCAACTGCATAAACTGGT
β-Actin (ACTB) F	*GACTTCGAGCAAGAGATGGC*
β-Actin (ACTB) R	ACAGGACTCCATGCCCAG
