# Supplementary figures and images for: PHGDH inhibition and FOXO3 modulation drives PUMA-dependent apoptosis in osteosarcoma
Source: Cell Death Dis. 2025 Feb 12;16(1):89. doi: 10.1038/s41419-025-07378-6 (PMC11814296; doi:10.1038/s41419-025-07378-6)

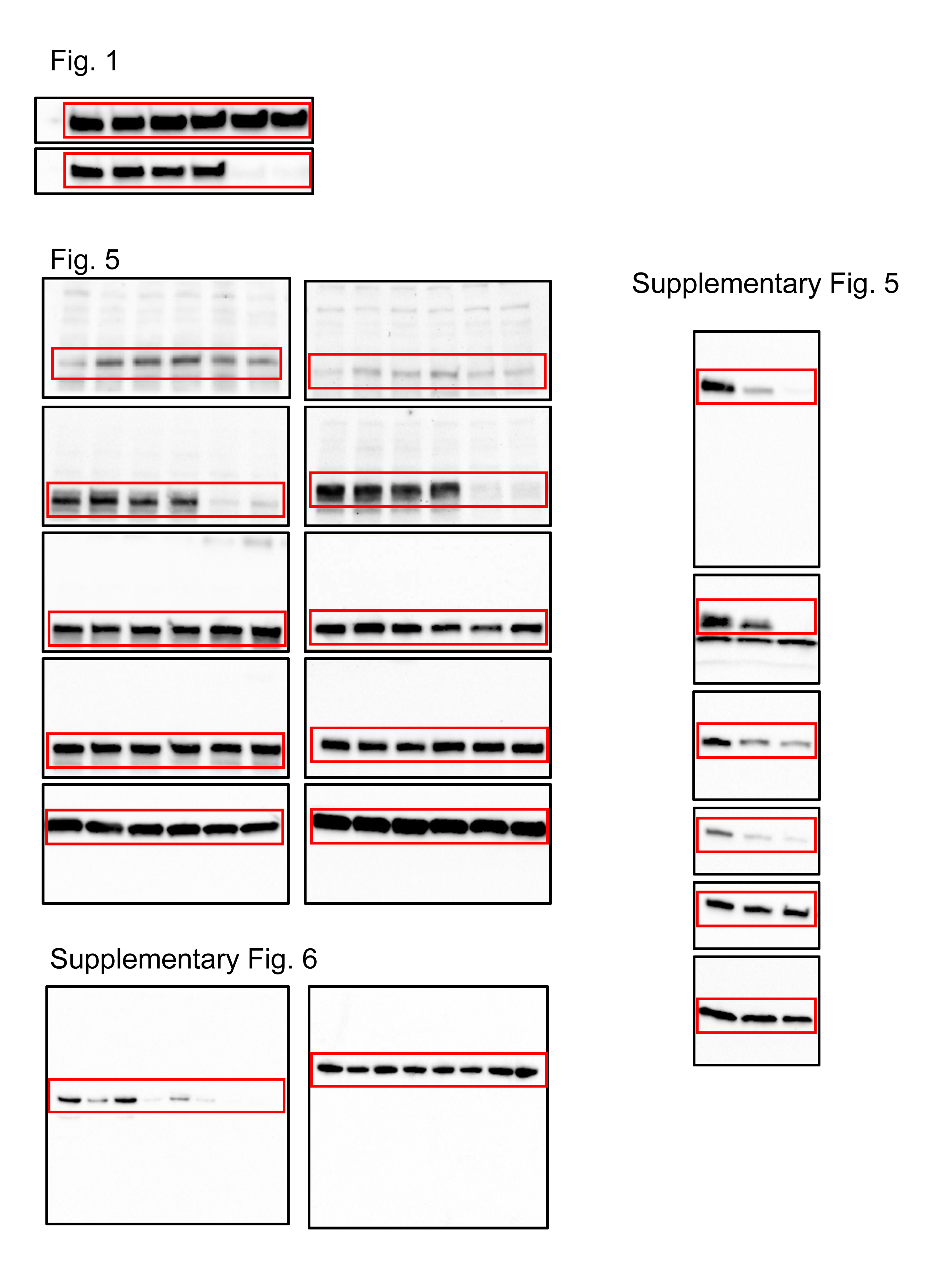

Supplement: Supplementary file 3 — Western raw data [file 41419_2025_7378_MOESM3_ESM.tif]
